# Dynamic modulations of urinary sphingolipid and glycerophospholipid levels in COVID-19 and correlations with COVID-19-associated kidney injuries

**DOI:** 10.1186/s12929-022-00880-5

**Published:** 2022-11-10

**Authors:** Makoto Kurano, Daisuke Jubishi, Koh Okamoto, Hideki Hashimoto, Eri Sakai, Yoshifumi Morita, Daisuke Saigusa, Kuniyuki Kano, Junken Aoki, Sohei Harada, Shu Okugawa, Kent Doi, Kyoji Moriya, Yutaka Yatomi

**Affiliations:** 1 Department of Clinical Laboratory Medicine, Graduate School of Medicine, The University of Tokyo, 7-3-1 Hongo, Bunkyo-Ku, Tokyo, 113-8655 Japan; 2 Department of Clinical Laboratory, The University of Tokyo Hospital, Tokyo, Japan; 3 Department of Infectious Diseases, Graduate School of Medicine, The University of Tokyo, Tokyo, Japan; 4 Laboratory of Biomedical and Analytical Sciences, Faculty of Pharma-Science, Teikyo University, Tokyo, Japan; 5 Department of Health Chemistry, Graduate School of Pharmaceutical Sciences, The University of Tokyo, Tokyo, Japan; 6 Department of Infection Control and Prevention, The University of Tokyo, Tokyo, Japan; 7 Department of Emergency and Critical Care Medicine, The University of Tokyo Hospital, Tokyo, Japan

**Keywords:** COVID-19-associated kidney injuries, Urine, Sphingolipids, Glycerophospholipids, Lipidomics

## Abstract

**Background:**

Among various complications of coronavirus disease 2019 (COVID-19), caused by severe acute respiratory syndrome coronavirus 2 (SARS-CoV-2), renal complications, namely COVID-19-associated kidney injuries, are related to the mortality of COVID-19.

**Methods:**

In this retrospective cross-sectional study, we measured the sphingolipids and glycerophospholipids, which have been shown to possess potent biological properties, using liquid chromatography-mass spectrometry in 272 urine samples collected longitudinally from 91 COVID-19 subjects and 95 control subjects without infectious diseases, to elucidate the pathogenesis of COVID-19-associated kidney injuries.

**Results:**

The urinary levels of C18:0, C18:1, C22:0, and C24:0 ceramides, sphingosine, dihydrosphingosine, phosphatidylcholine, lysophosphatidylcholine, lysophosphatidic acid, and phosphatidylglycerol decreased, while those of phosphatidylserine, lysophosphatidylserine, phosphatidylethanolamine, and lysophosphatidylethanolamine increased in patients with mild COVID-19, especially during the early phase (day 1–3), suggesting that these modulations might reflect the direct effects of infection with SARS-CoV-2. Generally, the urinary levels of sphingomyelin, ceramides, sphingosine, dihydrosphingosine, dihydrosphingosine l-phosphate, phosphatidylcholine, lysophosphatidic acid, phosphatidylserine, lysophosphatidylserine, phosphatidylethanolamine, lysophosphatidylethanolamine, phosphatidylglycerol, lysophosphatidylglycerol, phosphatidylinositol, and lysophosphatidylinositol increased, especially in patients with severe COVID-19 during the later phase, suggesting that their modulations might result from kidney injuries accompanying severe COVID-19.

**Conclusions:**

Considering the biological properties of sphingolipids and glycerophospholipids, an understanding of their urinary modulations in COVID-19 will help us to understand the mechanisms causing COVID-19-associated kidney injuries as well as general acute kidney injuries and may prompt researchers to develop laboratory tests for predicting maximum severity and/or novel reagents to suppress the renal complications of COVID-19.

**Supplementary Information:**

The online version contains supplementary material available at 10.1186/s12929-022-00880-5.

## Introduction

Coronavirus disease 2019 (COVID-19), caused by severe acute respiratory syndrome coronavirus 2 (SARS-CoV-2), is associated with various complications. Among them, renal complications, especially acute kidney injury (AKI), are associated with critical conditions. These renal complications are known as COVID-19-associated kidney injuries. A high incidence of AKI has been reported, especially among critically ill patients, and patients with COVID-19-associated kidney injuries reportedly have a higher risk of in-hospital death [7, 13, 14, 40]. Moreover, recent studies have suggested prolonged kidney dysfunction in some patients with COVID-19 [43].

Regarding the etiology of COVID-19-associated kidney injuries, the mechanisms have not been fully elucidated at present, and both direct and indirect mechanisms have been proposed [30]. ACE2 and TMPRSS2, which are key proteins for the entry of SARS-CoV-2 into human cells [15], are highly expressed in podocytes and proximal tubules in the kidney, and SARS-CoV-2 can directly infect and damage the kidney [6, 42, 47]. As indirect mechanisms, complications such as thrombosis and endotheliitis, which are frequently observed in COVID-19 patients [57, 62, 64], can cause renal impairment. In addition, other mechanisms not specific to COVID-19, such as right heart failure [5], cytokines, and nephrotoxins [30], have been proposed to be involved in COVID-19-associated kidney injuries.

Since COVID-19-associated kidney injuries are important in terms of clinical outcomes and the underlying mechanisms have yet to be fully elucidated, as described above, investigating biomarkers of COVID-19-associated kidney injuries will be important to understand the pathogenesis of such complications. Urine samples rapidly and accurately reflect renal conditions. Actually, recent studies have revealed that urinary chemical biomarkers (urinary total protein [TP], *N*-acetyl-β-d-glucosaminidase [NAG], α1-microglobulin [α1-MG], neutrophil gelatinase-associated lipocalin [NGAL], and liver type fatty acid-binding protein [L-FABP]) and urine sediment findings are correlated with both the severity of COVID-19 and COVID-19-associated kidney injuries [16, 19, 35]. Therefore, in the present study, we investigated urinary biomarkers for COVID-19-associated kidney injuries.

In this study, we focused on sphingolipids and glycerophospholipids. A series of basic and clinical studies have revealed the importance of these lipids in the pathogenesis of various human diseases including kidney diseases. Among the sphingolipids, the bioactivities of sphingosine 1-phosphate (S1P) and ceramides have been well studied. S1P possesses potent anti-apoptotic and pro-survival properties [27, 33]. A series of basic studies showed that the S1P1 signal might attenuate both acute and chronic kidney diseases through its pro-survival, anti-inflammation, anti-fibrosis, and vasoprotective properties [1, 12, 24, 25, 50], whereas the S1P2 signal might aggravate kidney disease by accelerating fibrosis and inflammation [24, 49]. Ceramides have both pro-apoptosis and pro-inflammation properties [44, 61] and have been shown to accelerate the pathological condition of both chronic and acute kidney diseases in basic studies [41, 56]. Regarding the metabolism of sphingolipids, ceramides are derived from sphingomyelin (SM) and can be converted into sphingosine (Sph). S1P is produced from Sph by S1P kinases [33]. Dihydrosphingosine 1-phosphate (dhS1P), another analog for S1P receptors, is produced from dihydrosphingosine (dhSph) by S1P kinases, and dhSph is processed into ceramides via dihydroceramides [2]. In clinical studies, urinary ceramide levels were reportedly associated with diabetic nephropathy [38, 51], and urinary SM levels are altered in chronic kidney diseases [67]. However, the modulation of urinary sphingolipid levels remains unknown, especially in terms of the pathogenesis of human AKI.

Among glycerophospholipids, lysophosphatidic acids (LPA) and lysophosphatidylcholine (LPC) have been well studied in the fields of nephrology. LPA is produced from LPC by autotaxin, and six kinds of LPA receptors have been identified [68]. The roles of LPA in inflammation depend on its receptors. LPA can exacerbate the pathogenesis of chronic kidney diseases, resulting in inflammation and fibrosis [29, 53], while it can also reportedly protect against acute kidney diseases [9, 34]. The urinary LPA levels are elevated in diabetic nephropathy [55], while the urinary autotaxin levels increase in membranous nephropathy [36]. The urinary LPC levels are also positively correlated with kidney dysfunction, and LPC itself might exert lipotoxicity [69]. Lysophosphatidylinositol (LPI) reportedly exacerbates the pathogenesis of sepsis-associated AKI [22]. Regarding other glycerolysophospholipids, such as lysophosphatidylethanolamine (LPE), lysophosphatidylglycerol (LPG), and lysophosphatidylserine (LPS), their roles in kidney injuries remain unknown, and the modulation of urinary lysophospholipids in AKI also remains to be elucidated. Along with LPA, the GPR34, P2Y10, and GPR174 receptors have been shown to be specific for LPS [17], while the GPR55 receptor is specific for LPI and LPG [45]. LPC, LPS, LPE, LPI, and LPG are produced from phosphatidylcholine (PC), phosphatidylserine (PS), phosphatidylethanolamine (PE), phosphatidylinositol (PI), and phosphatidylglycerol (PG), respectively. Although only a limited number of studies are available, PC has been shown to possess protective effects against acute kidney injuries through its antioxidant properties [10, 28], and PS might reduce nephrotoxicity by suppressing inflammation [21]. The roles of other diacylphospholipids in the pathogenesis of AKI have not been reported. Regarding the modulations of urinary diacylphospholipids in humans, a recent study showed that urinary PC levels might be associated with adverse outcomes and mortality in patients with chronic kidney diseases [60]; however, their associations with human AKI are not well known at present.

Although several lipidomics analyses using serum or plasma samples from patients with COVID-19 have been performed [23], only one lipidomics study has been conducted using urine samples from a very small number of COVID-19 patients [31]. With this background in mind, we performed lipidomic analyses using urine samples to investigate the mechanisms responsible for COVID-19-associated kidney injuries to understand the pathogenesis of COVID-19 and COVID-19-associated kidney injuries, as well as AKI, better and to help researchers develop reagents capable of preventing severe kidney injuries in the future. In this study, we measured the longitudinal urinary levels of sphingolipids and glycerophospholipids in 272 samples from 91 COVID-19 subjects and 95 samples from 95 control subjects without infectious disease.

## Methods

### Samples

We collected the residual urinary samples after routine clinical testing from 91 subjects who had been diagnosed as having COVID-19 using an RT-PCR assay between September 2020 and April 2021. The sampling times were classified into the following eight periods: day 1–3, day 4–6, day 7–9, day 10–12, day 13–15, day 16–18, day 19–24, and day 25–40 after symptom onset. Since the timing of RT-PCR testing varied largely among the patients, we used the day after symptom onset as the initial measurement in our investigation of lipid modulation. None of the subjects enrolled in the present study had been vaccinated at the time of sampling.

The subjects were classified into three groups according to the maximum severity of COVID-19: maximum severity group 1 (did not require oxygen supplementation), maximum severity group 2 (required oxygen supplementation, but did not require mechanical ventilatory support), and maximum severity group 3 (required mechanical ventilatory support). As a control, we collected 95 urine samples from volunteers without infectious diseases.

The current study was performed in accordance with the ethical guidelines established by the Declaration of Helsinki. Written informed consent for sample analysis was obtained from some of the patients. For the remaining participants from whom written informed consent could not be obtained (because they had been discharged or transferred out of the hospital), informed consent was obtained in the form of an opt-out on our institution’s website, as follows. Patients were informed of the study through the website, and those who were unwilling to be enrolled were excluded. The study design was approved by The University of Tokyo Medical Research Center Ethics Committee (2602 and 2020206NI).

### Measurement of glycerolysophospholipids, diacylphospholipids, and sphingolipids using LC–MS/MS

We measured the levels of the lipid mediators listed below using four independent LC–MS/MS methods and the LC8060 system, consisting of a quantum ultra-triple quadrupole mass spectrometer (Shimadzu, Japan). We simultaneously measured six ceramide species (Cer d18:1/16:0 [C16:0], Cer d18:1/18:0 [C18:0], Cer d18:1/18:1 [C18:1], Cer d18:1/20:0 [C20:0], Cer d18:1/22:0 [C22:0], and Cer d18:1/24:0 [C24:0]), Sph, and dhSph, as previously described [38]. We also measured S1P and dhS1P, as described previously [52]. Furthermore, LPA, LPC, LPS, LPI, LPG, and LPE were also measured, as described previously [37]. We monitored 11 acyl chains (14:0, 16:0, 16:1, 18:0, 18:1, 18:2, 18:3, 20:3, 20:4, 20:5, and 22:6) for these lysophospholipids as well as 22:5 LPI. We also measured SM and diacylphospholipids, including PC, PE, PI, PG, and PS [26]. We monitored 17 diacyl chains (32:1, 32:2, 34:1, 34:2, 36:1, 36:2, 36:3, 36:4, 38:1, 38:2, 38:3, 38:4, 38:5, 38:6, 40:1, 40:2, and 40:7) for SM and 64 diacyl chains (28:0, 28:1, 28:2, 30:0, 30:1, 30:2, 32:0, 32:1, 32:2, 32:3, 32:4, 34:0, 34:1, 34:2, 34:3, 34:4, 34:5, 34:6, 36:0, 36:1, 36:2, 36:3, 36:4, 36:5, 36:6, 36:7, 38:0, 38:1, 38:2, 38:3, 38:4, 38:5, 38:6, 38:7, 38:8, 40:0, 40:1, 40:2, 40:3, 40:4, 40:5, 40:6, 40:7, 40:8, 40:9, 40:10, 42:0, 42:1, 42:2,42:3, 42:4, 42:5, 42:5, 42:6, 42:7, 42:8, 42:9, 42:10, 42:11, 44:0, 44:1, 44:2, 44:6, 44:7, and 44:12) for PC, PE, PI, PG, and PS. With the exceptions of SM and the diacylphospholipids, both the intra-day and inter-day coefficients of variation for the metabolites were below 20%, as validated in our previous papers [37, 38, 52]. The urinary levels of the measured lipids were adjusted to the urinary creatinine levels.

### Urinalysis

To measure the urinary clinical markers, we used the reagents as described previously [35]. Renal tubular epithelial cells (RTE) were counted per high-power field of view (/HPF); urinary casts were classified into hyaline casts (HyaC), granular casts (GraC), epithelial casts (RTEC), and waxy casts (WaxC), and their numbers were counted per whole field (/WF). The RTE findings were classified as rank 0 (absent), rank 1 (< 1/HPF), rank 2 (1–4/HPF), rank 3 (5–9/HPF), or rank 4 (> 10/HPF), those of HyaC were classified as rank 0 (absent), rank 1 (< 4/WF), rank 2 (5–19/WF), rank 3 (20–49/WF), or rank 4 (> 50/WF), those of GraC and RTEC were classified as rank 0 (absent), rank 1 (< 4/WF), rank 2 (5–49/WF), or rank 3 (> 50/WF), and those of WaxC were classified as rank 0 (absent) or rank 1 (present).

### Statistical analysis

The data were analyzed using SPSS (Chicago, IL) or MetaboAnalyst (https://www.metaboanalyst.ca/). To examine differences in the time courses of urinary lipids among the control subjects and maximum severity groups 1, 2, and 3, we evaluated significant differences using the Kruskal–Wallis test, followed by the Steel–Dwass test as a post-hoc test. To examine differences in the urinary lipid levels longitudinally between specific time points in a specific maximum severity group, we used the paired Wilcoxon signed-rank test. To examine differences between the control subjects and the COVID-19 subjects, we performed non-parametric Volcano plot analyses. For the correlation studies, a Kendall rank correlation was performed to examine the correlations of lipids and clinical data with the maximum severity of COVID-19, using age, sex, and the presence of diabetes, hypertension, and current smoking as covariates of interest. To construct machine learning models, we used SPSS modeler ver. 18:2 (Chicago, IL) and performed CHAID analyses, SVM analyses, and neural network analyses. The Spearman rank correlation was performed to examine correlations between lipids and clinical data. The independent effects of the clinical properties and the results of urinary laboratory tests on urinary lipid levels were investigated with a stepwise multiple regression analysis, using urinary lipid levels as objective variables and clinical information, maximum severity, eGFR, CRP, d-Dimer, urinary chemical markers, urinary sediment findings, SG, pH, and urinary sodium levels as possible explanatory factors. To examine differences between the subjects treated with antiviral reagents and those without, we used the Mann–Whitney U test. The graphs shown in the figures were prepared using Graphpad Prism 9 (GraphPad Software, San Diego, CA) or MetaboAnalyst. *P* values of less than 0.05 were deemed as denoting statistical significance in all the analyses.

## Results

### Characteristics of the subjects and the analyzed samples

The characteristics of all the subjects and the numbers of samples analyzed at each specific time point are described in Additional file 1: Tables S1 and S2, respectively. As shown in the tables, differences in patient age were seen among the maximum severity groups, while differences in the percentage of patients with hypertension were seen between the control subjects and the maximum severity groups. We also observed differences in sex between the control subjects and the maximum severity groups on day 19–24 and day 25–40. In the control group, no differences in the sphingolipid and total glycerophospholipid levels were seen between subjects with hypertension and those without hypertension. Therefore, the presence of hypertension might not have had a large effect on the results in the present analyses. Regarding sex, the urinary levels of several monitored lipids were higher in female subjects. The ratios of the lipid levels in female subjects relative to those in male subjects were 140.3% for the total SM levels, 191.2% for the S1P levels, 154.0% for the dhS1P levels, 186.6% for the Sph levels, 152.1% for the dhSph levels, 150.9% for the C18:1 Cer levels, 141.1% for the total LPG levels, 145.5% for the total PC levels, 144.2% for the total PE levels, 165.6% for the total PG levels, 151.5% for the total PI levels, and 125.1% for the total PS levels. Age was positively correlated with the total LPC levels (*r* = 0.214, *p* = 0.037) and the total PS levels (*r* = 0.218, *p* = 0.034). Therefore, we think that these correlations were thought to have had a minimal impact on the interpretation of the dynamic modulations of the monitored lipid levels, as described below.

The time courses for the urinalysis results and other clinical parameters are shown in Additional file 1: Fig. S1. Overall, the modulations of these parameters seemed reasonable, while a remarkable decline in eGFR was not observed in the COVID-19 subjects.

### Urinary sphingolipids generally decreased in mild COVID-19 during the early phase, while they generally increased in severe COVID-19, with the exception of C18:0 Cer

**Fig. 1:**
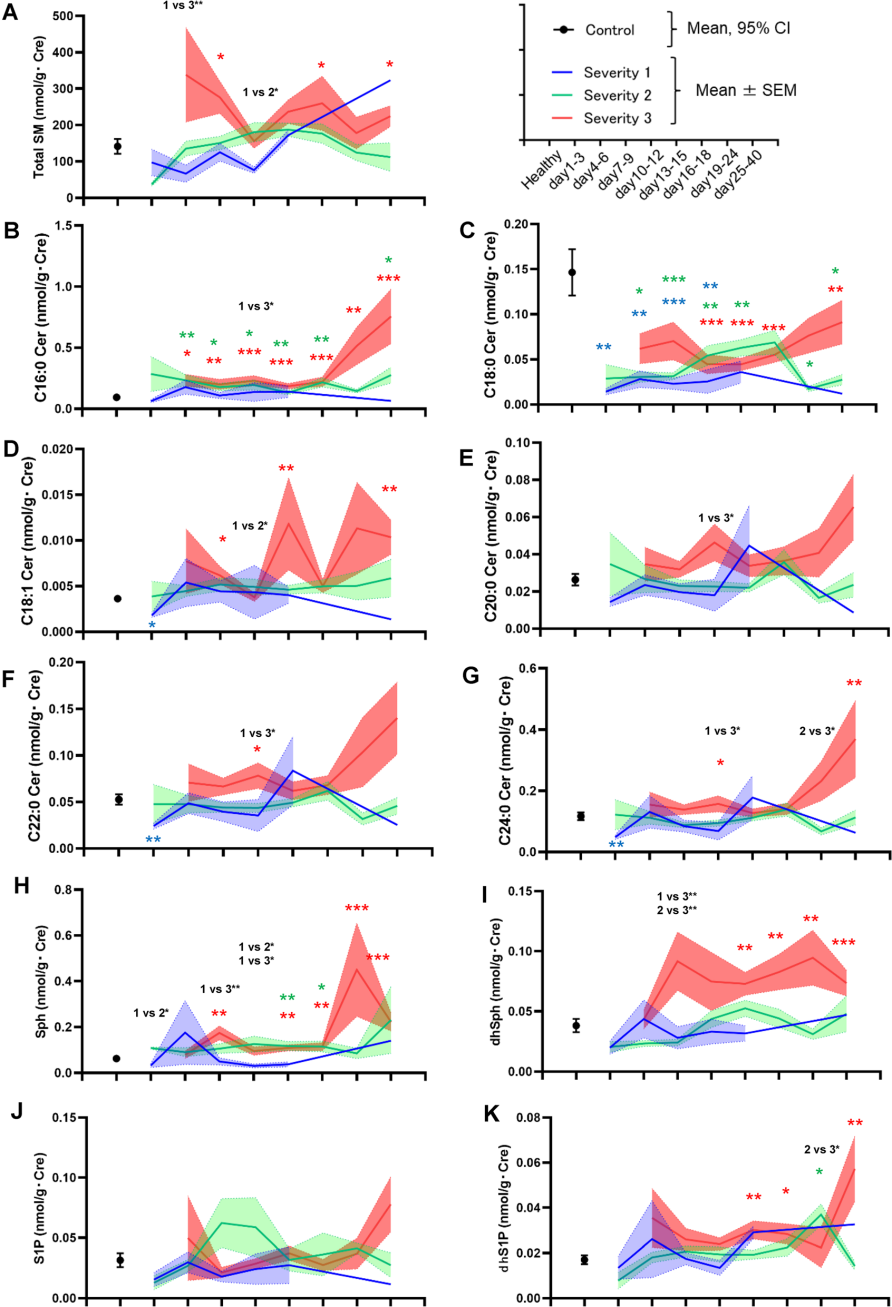
Modulations of urinary sphingolipid levels during the time course of COVID-19. The urinary sphingolipid levels were measured longitudinally in symptomatic COVID-19 subjects (n = 91). The significance of the Steel–Dwass test followed by the Kruskal–Wallis test for analyses of healthy subjects and the maximum severity groups (defined in the Methods) is shown as *p < .05, **p < .01, or ***p < .001 for comparisons with healthy subjects or between maximum severity groups. The ranges for the control subjects (n = 95) are shown as the 95% confidence interval (CI), while those for the maximum severity groups are shown as the mean ± SEM

Figure 1 shows the time courses of the urinary sphingolipid levels in the COVID-19 subjects. C16:0 Cer and SM increased most rapidly from day 4–6. C18:1 Cer, C20:0 Cer, C22:0 Cer, C24:0 Cer, Sph, dhSph, and dhS1P significantly decreased or tended to decrease during the early phase (day 1–3) in maximum severity group 1 and then increased, especially in maximum severity group 3. Among the monitored sphingolipids, the C18:0 Cer levels rapidly decreased from day 1–3. The urinary levels of several sphingolipids seemed remarkably higher on day 25–40; however, several biases might be present, since samples were collected only from patients with severe COVID-19 who were still hospitalized on day 25–40. Regarding longitudinal comparisons, although we could compare the urinary lipid levels between only limited time points, the results showed the elevation of urinary sphingolipids during the time course of COVID-19, especially in day 19–40 in maximum severity group 3 (Additional file 1: Figs. S2A-D and S3).

### Urinary glycerophospholipids generally increased in severe COVID-19, depending on maximum severity and time course

**Fig. 2:**
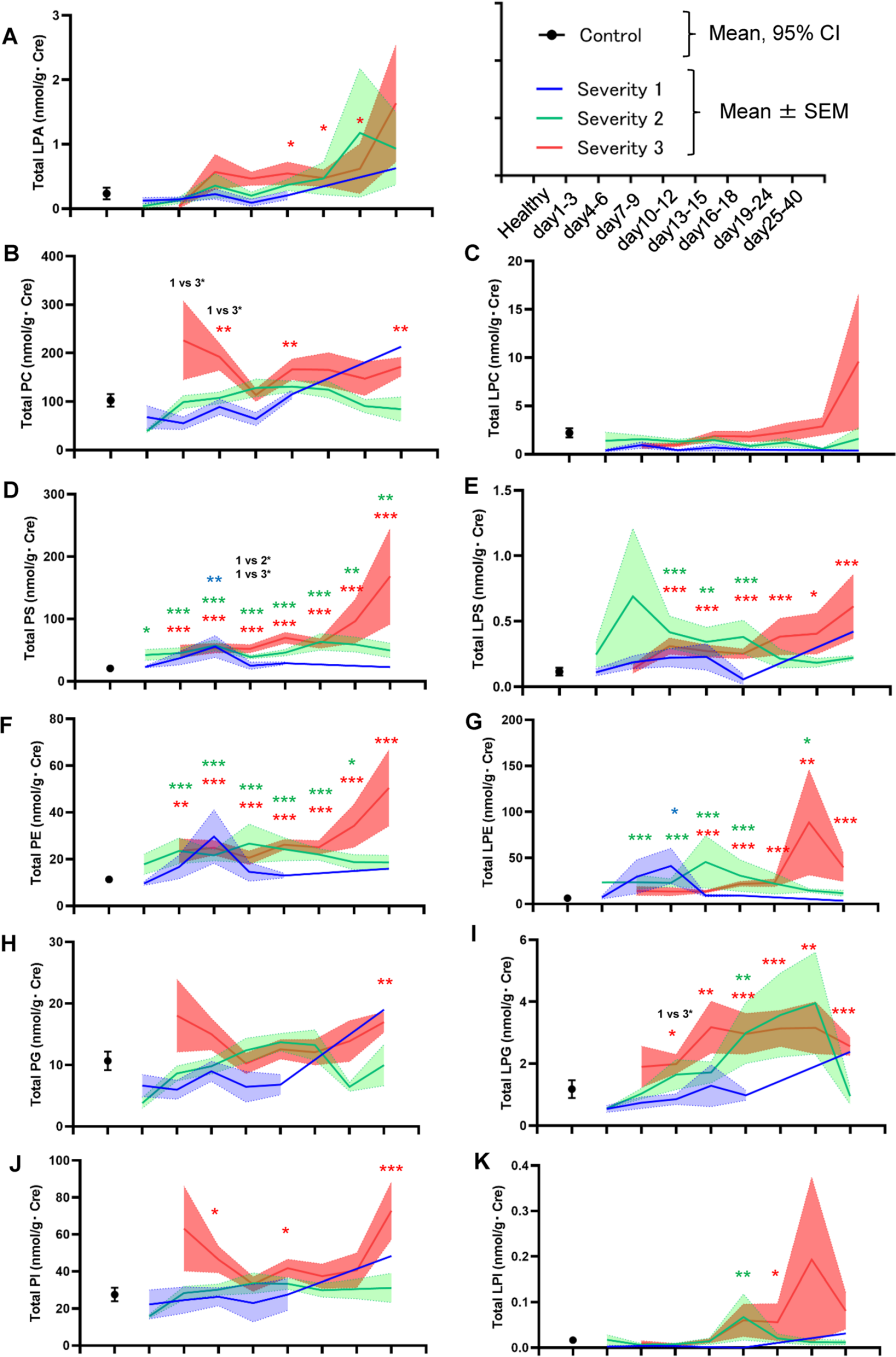
Modulations of urinary glycerophospholipid levels during the time course of COVID-19. The urinary glycerophospholipid levels were measured longitudinally in symptomatic COVID-19 subjects (n = 91). The significance of the Steel–Dwass test followed by the Kruskal–Wallis test for analyses of healthy subjects and the maximum severity groups (defined in the Methods) is shown as *p < .05, **p < .01, or ***p < .001 for comparisons with healthy subjects or between maximum severity groups. The ranges for the control subjects (n = 95) are shown as the 95% confidence interval (CI), while those for the maximum severity groups are shown as the mean ± SEM

Figure 2 shows an overview of the total glycerophospholipid modulations. The total levels of all the monitored glycerophospholipids increased, especially in severe COVID-19. Regarding the PC-LPC-LPA axis, the urinary PC levels rapidly increased in maximum severity group 3; they tended to decrease in maximum severity groups 1 and 2. The urinary LPA and LPC levels increased, especially during the later phase. Regarding the PS-LPS axis, PS increased rapidly from the early phase (day 1–3) and LPS increased from day 7–9, especially in patients with severe COVID-19. Regarding the PE-LPE axis, LPE increased from the early phase (day 4–6) and reached a peak on day 10–12 in maximum severity group 2. In maximum severity group 3, LPE increased, especially during the later phase (day 19–24). The urinary total PE levels were modulated in an almost similar manner to those of LPE. Regarding the PG-LPG axis and the PI-LPI axis, LPG and LPI increased in maximum severity groups 2 and 3 from around the middle phase (day 7–15), while PG and PI increased only in maximum severity group 3. Regarding longitudinal comparisons, the paired statistical analyses showed the elevation of urinary glycerophospholipids during the time course of COVID-19, especially in day 19–40 in maximum severity group 3 (Additional file 1: Fig. S2E-H, S4).

### C18:0 Cer was maintained at lower levels throughout the time course, while PC and LPC decreased especially during the early phase and PE and PS increased especially during the later phase

**Fig. 3:**
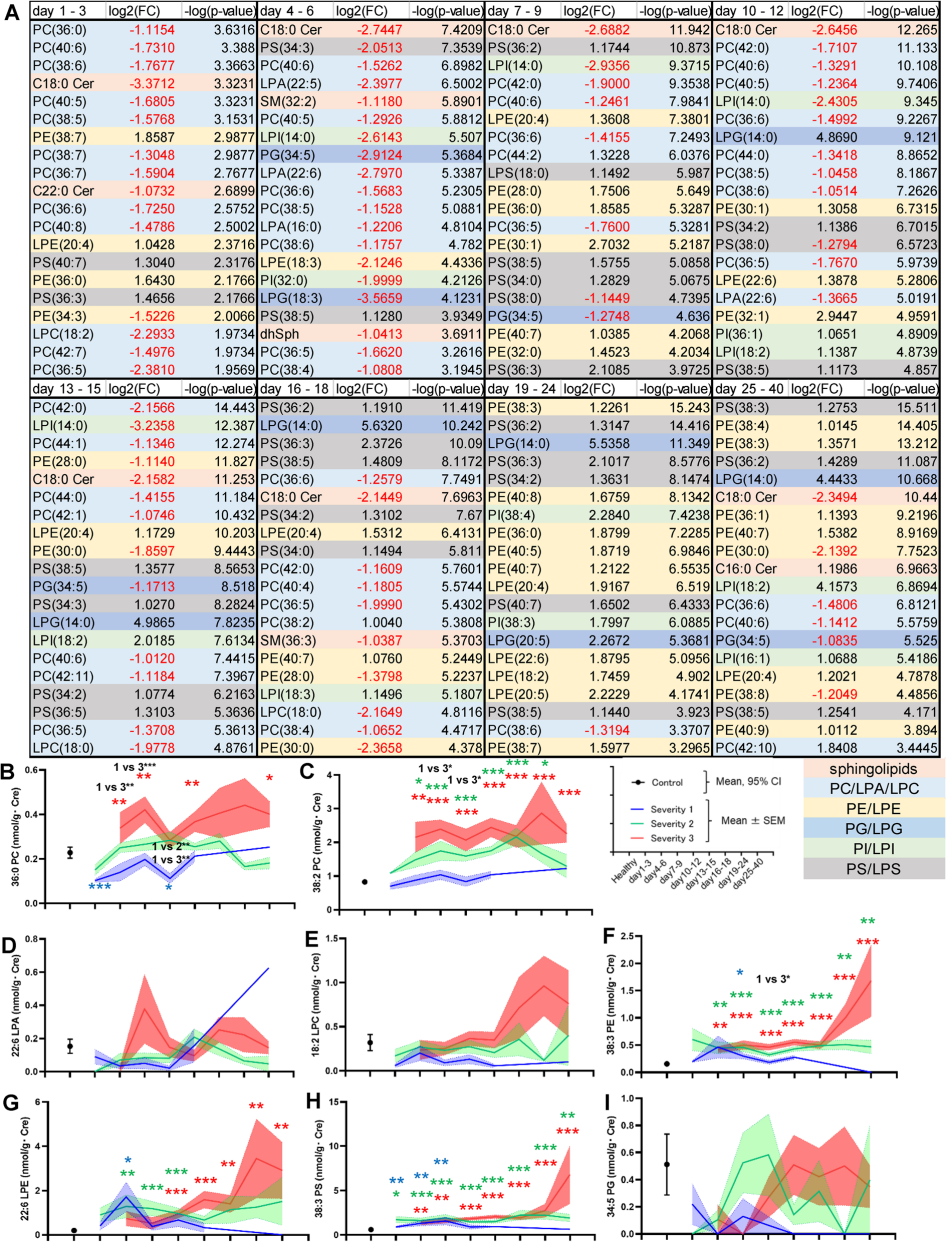
Time course of volcano plot analyses for largely modulated lipid species in COVID-19. The volcano plot analyses were performed using samples collected from control subjects and COVID-19 subjects at the specific time points shown in Additional file 1: Table S2. **A** Log_2_(FC) and *p* values of the lipids with 20 lowest *p* values. **B**–**I** Time courses of the representative lipids in **A** shown in a manner similar to that used in Figs. 1 and 2

To investigate time-course-dependent lipid modulations in greater detail, we next created separate volcano plots for each sampling point, as shown in Additional file 1: Figs. S5–S8. To understand the lipid modulations that occur in patients with COVID-19 better, the lipids with the 20 lowest *p* values at each time period were selected; their log_2_(FC) and *p* values are shown in Fig. 3A. Among the sphingolipids, the C18:0 Cer level decreased markedly, especially during the early phase. Decreased levels of PC, LPA, and LPC species were clearly observed until day 16–18, while the levels of several species including 38:2 PC, 42:10 PC, and 44:2 PC increased. Regarding the PE-LPE axis, increases in PE and LPE species were clearly observed after day 7–9, especially on day 19–24 and day 25–40. The levels of several species including 28:0 PE, 30:0 PE, 34:3 PE, 38:8 PE, and 18:3 LPE decreased. Regarding the PS-LPS axis, specific PS species, such as 36:2 PS and 36:3 PS, increased, especially during the middle phase (day 16–18 and day 19–24), while 38:5 PS consistently increased almost throughout the time course. Decreases in 38:0 PS and 34:3 PS were observed on day 7–12 and day 4–6, respectively. 18:0 LPS increased on day 7–9. Regarding the PI-LPI axis, 14:0 LPI decreased on day 4–15 and 32:0 PI decreased on day 4–6, while 16:1 LPI, 18:2 LPI, and 18:3 LPI and several PI species containing 16:1, 18:1, and 18:3 acyl chains increased after day 10–12. Regarding the PG-LPG axis, the increase in 14:0 LPG after day 10–12 and the decrease in 34:5 PG throughout the time course seemed characteristic. The time courses of the representative lipids are shown in Fig. 3B–I and Additional file 1: Figs. S9, S10.

### In general, SM and PC were positively correlated, and PE, PG, PI, and PS were negatively correlated with maximum COVID-19 severity

**Fig. 4:**
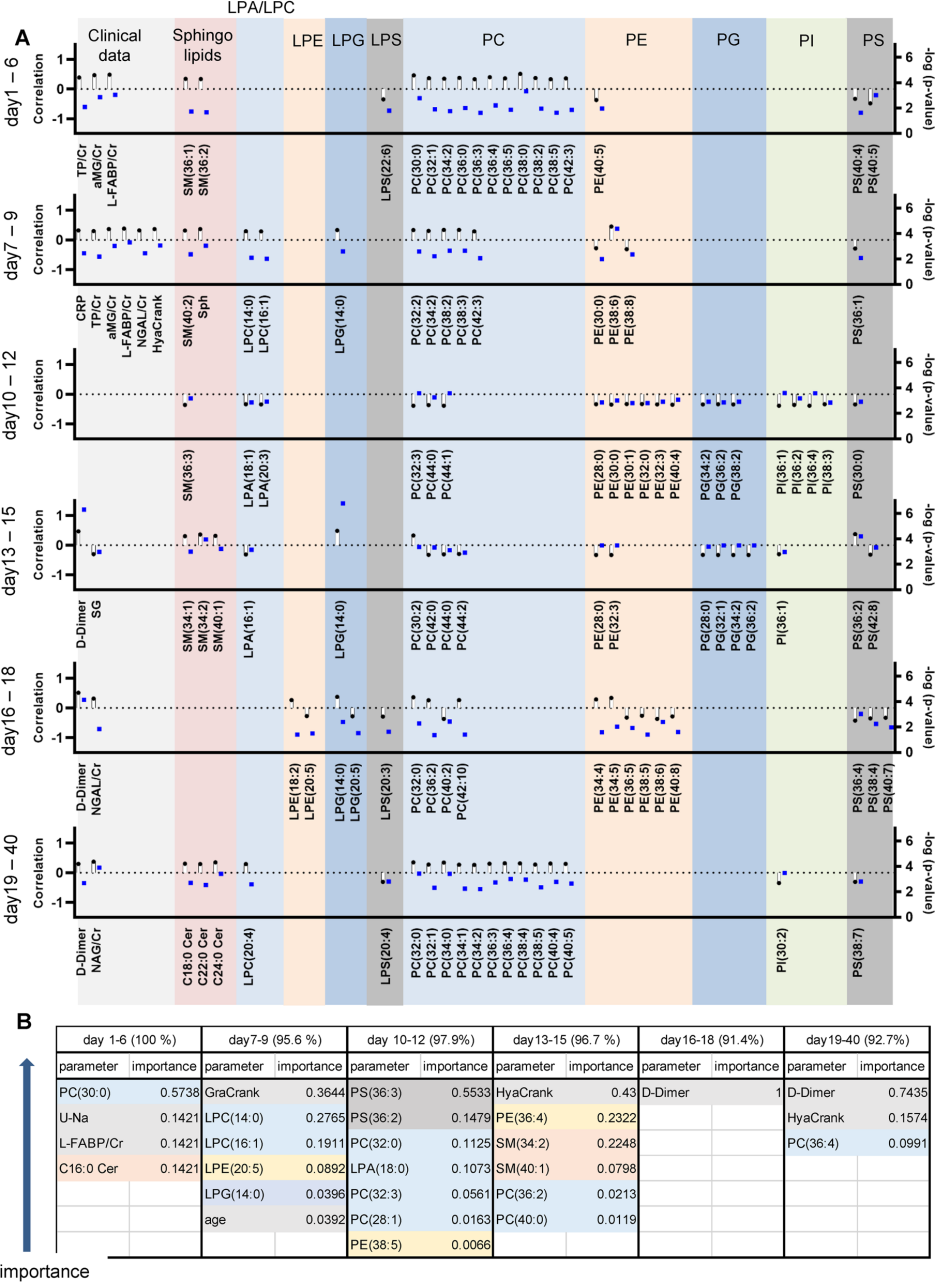
Correlations of monitored urinary lipid levels with maximum severity of COVID-19. Kendall rank correlation analyses were used to compare lipids or clinical parameters and the maximum severity of COVID-19, considering age, sex, and the presence of diabetes, hypertension, and current smoking as covariates of interest. **A** Time courses of the correlation coefficients and *p* values (shown as − log_10_ [*p* value]) at specific time points for the lipids and clinical parameters with the 20 lowest *p* values. **B** Lipids or clinical parameters selected using CHAID analyses as having a high importance for the construction of a machine learning model capable of predicting maximum severity at a specific time point

Next, we performed correlation analyses with the maximum severity of COVID-19, using age, sex, the presence of diabetes and hypertension, and current smoking as covariates of interest. Figure 4A shows the correlation coefficients and the *p* values of the lipids and clinical parameters with the 20 lowest *p* values at any specific time points. Among sphingolipids, SM (except for 36:3 SM) and Sph were positively correlated with maximum severity on day 10–12. On day 19–40, C18:0 Cer, C22:0 Cer, and C24:0 Cer were positively correlated with maximum severity. Regarding LPA, the 18:1 LPA, 20:3 LPA, and 16:1 LPA levels on day 10–12 and day 13–15 were negatively correlated with maximum severity. The 14:0 LPC and 16:1 LPC levels on day 7–9 and the 20:4 LPC levels on day 19–40 were positively correlated with maximum severity. Many PC species had strong positive correlations with maximum severity, while the 32:3 PC, 40:2 PC, 42:0 PC, 44:0 PC, 44:1 PC, and 44:2 PC levels during the middle phase (day 10–18) had rather strong negative correlations with maximum severity. Many PE species were negatively correlated with maximum severity, while 34:4 PE, 34:5 PE, and 38:6 PE at some sampling points were positively correlated with maximum severity. Several PG and PI species, especially on day 10–15, were negatively correlated with maximum severity. Among LPG species, 14:0 LPG was positively correlated with maximum severity. Meanwhile, LPS and PS species were, in general, negatively correlated with maximum severity. Additional file 1: Fig. S11 shows the time courses of characteristic lipids.

### PC-LPA-LPC axis throughout the time course, sphingolipids during the early phase, and PS and PE-LPE axis during the middle phase were important in machine learning models for predicting maximum severity

In addition to performing simple correlation studies, we investigated which lipids and clinical parameters were strongly correlated with the maximum severity of COVID-19 using machine learning techniques. Figure 4B and Additional file 1: Fig. S12A, B show the lipids or clinical parameters selected with high importance by CHAID analyses, SVM analyses, and neural network analyses, respectively.

In the CHAID analyses, the PC-LPA-LPC axis throughout the time course, except day 16–18, had a high importance for determining maximum severity in the constructed models. The PE-LPE axis on day 7–15, PS on day 10–12, C16:0 Cer on day 1–6, SM on day 13–15, and 14:0 LPG on day 7–9 had some importance. In the SVM and neural network analyses, although the importance of each parameter was relatively low, the PC-LPA-LPC axis throughout the time course, sphingolipids during the early phase, and PS and the PE-LPE axis during the middle phase had importance for determining maximum severity in the constructed models.

### Sphingolipids, except SM and dhS1P, LPC, PS, LPS, PE, LPE were positively associated with both the urinary chemical markers and the urinary sediment findings

**Fig. 5:**
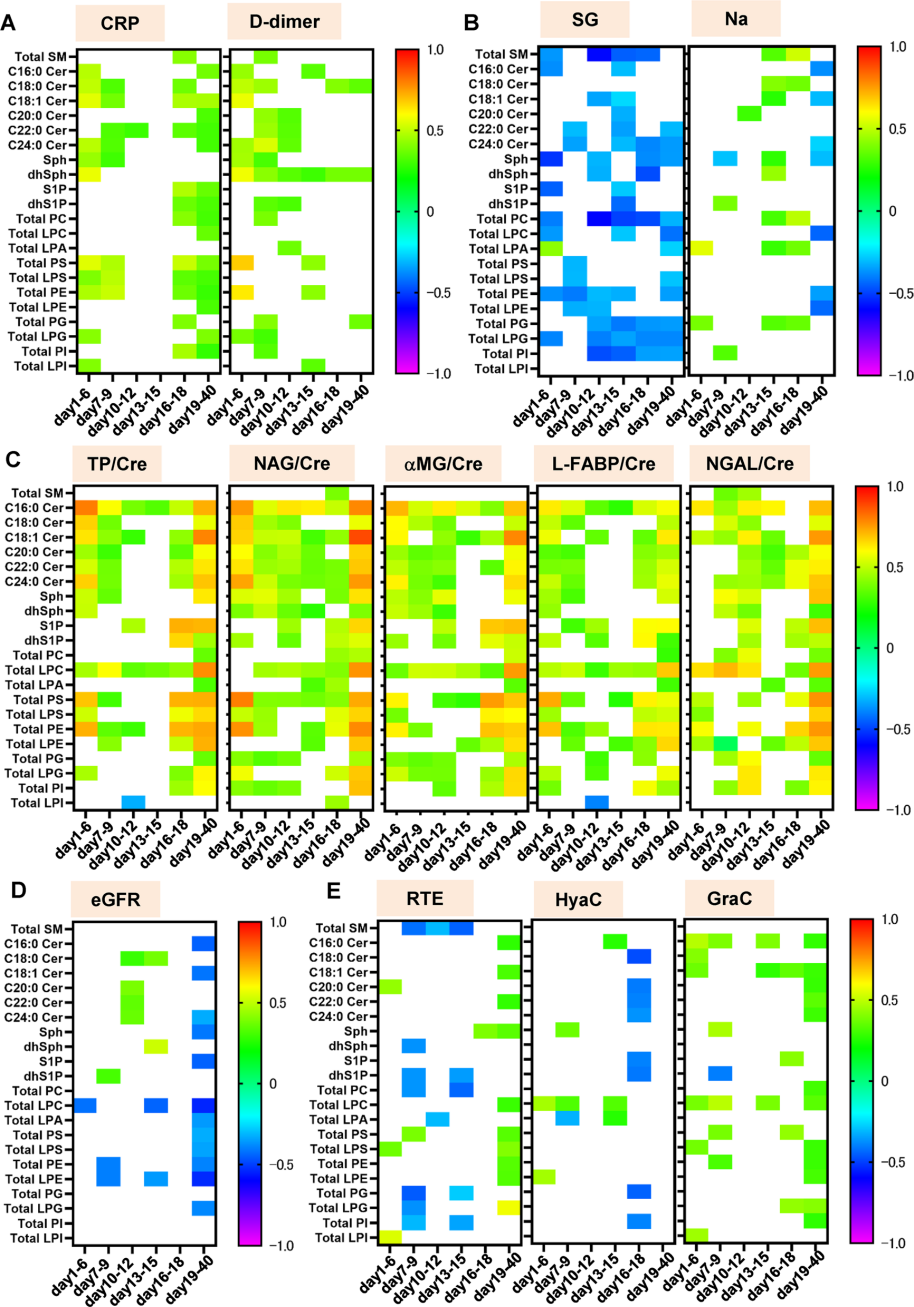
Correlations of monitored lipids with clinical parameters. Spearman rank correlation analyses were used to compare the urinary levels of sphingolipids and the total levels of glycerophospholipids with various clinical parameters including serum CRP, and d-dimer levels (**A**), SG and urinary sodium levels (**B**), urinary chemical markers (**C**), eGFR (**D**), and urinary sediment findings (**E**) in samples collected at the specific time points. Correlation coefficients are shown as a heat map

Next, we investigated the correlations of the monitored lipids with clinical parameters. Figure 5 and Additional file 1: Figs. S13, S14 shows the time courses of the correlations. As shown in Fig. 5A, the urinary levels of several lipids had positive correlations with serum CRP and d-Dimer levels. The lipids which had a negative correlation with the urinary SG levels and a positive correlation with sodium levels are deemed to increase in the pathogenesis the renal factors or decrease in the pathogenesis of prerenal kidney injuries. As shown in Fig. 5B, the urinary SM, C18:1 Cer, PC, and PG levels had negative correlations with the urinary SG levels and positive ones with the urinary sodium levels in the middle phase (day 13–18).

The urinary TP levels were positively correlated with ceramides, Sph, LPC, PS, LPS, PE, LPE, and LPG in the early phase (day 1–6 and/or day 7–9) and in the late phase (day 16–18 and/or day 19–40). They consistently had positive correlations with the urinary C16:0 Cer and LPC levels. Regarding the urinary chemical biomarkers, generally, urinary sphingolipids except SM had positive correlations with urinary chemical biomarkers. Especially, the C16:0 and C18:1 ceramides had positive correlations almost throughout the monitored periods. The urinary glycerophospholipids, except PC, LPA, and LPI, generally had positive correlations with the urinary chemical markers (Fig. 5C). Interestingly, the eGFR levels were positively correlated with the urinary levels of several sphingolipids, while they were negatively correlated with the urinary LPC, LPE, and PE levels in the early to the middle phase. In the late phase (day 19–40), the eGFR levels were negatively correlated with the urinary sphingolipids and glycerophospholipids (Fig. 5D).

Regarding the urinary sediment findings, the urinary SM and dhS1P levels were negatively correlated with RTE, while the urinary ceramides levels were positively correlated with RTE and GraC, except the negative correlations observed in day 16–18. Among glycerophospholipids, the urinary PC, LPA, PG, LPG, PI levels had negative correlations with RTE in day 7–15. The urinary LPC levels had positive correlations with the urinary sediment findings in many time points. The urinary PS, LPS, PE, and LPE also had positive correlations with the urinary sediment findings in the early phase (day 1–9) (Fig. 5E and Additional file 1: Fig. S14).

We further investigated the independent effects of the systematic severity of COVID-19, represented by d-Dimer and CRP, and renal injuries, represented by the results of urinary laboratory tests on urinary lipid levels, were evaluated with a multiple regression analysis, using urinary lipid levels as subjective variables. As shown in Additional file 1: Figs. S15–S18, urinary chemical makers such as NGAL and L-FABP were selected as positive explanatory factors with high β values for ceramides, S1P, dhS1P, dhSph, LPC, LPS, LPE, LPG, LPA, PC, PE, PG, PI, and PS, whereas d-Dimer or maximum severity were selected as positive explanatory variables for SM, dhSph, LPI, when all the samples were analyzed. Although, when samples were analyzed separately according to the days after the onset of COVID-19, the results were not always consistent, these results suggested that both renal injuries and systematic severity would affect the dynamic modulations of urinary sphingolipids and glycerophospholipids in COVID-19.

In addition, although, since this is a cross-sectional study, we could not conclude the possible influences of antiviral therapy on urinary lipid levels, we also observed some differences in urinary lipid levels between subjects treated with antiviral reagents such as remdesivir [63] and favipiravir [11] and those without (Additional file 1: Figs. S19, S20).

### Validation of the modulations of urinary sphingolipid and glycerophospholipid levels in COVID-19 in independent samples

Lastly, after we finished all the analyses, to validate the main results, we measured 46 additional urine samples collected from 31 independent subjects who had been diagnosed as having COVID-19 using an RT-PCR assay between April 2021 and August 2021. Additional file 1: Figs. S21 and S22 show the concentrations of lipids, overlayed on Figs. 1 or 2. As shown in these figures, the modulations of sphingolipids and glycerophospholipids were generally replicated. Moreover, when we investigate the accuracy of the predicting models for maximum severity described in Fig. 4B and Additional file 1: Fig. S12, using these independent samples, we obtained the accuracy of over 80% (Additional file 1: Table S3). Considering these results, we think that the modulations of these lipids could be replicated.

## Discussion

**Fig. 6:**
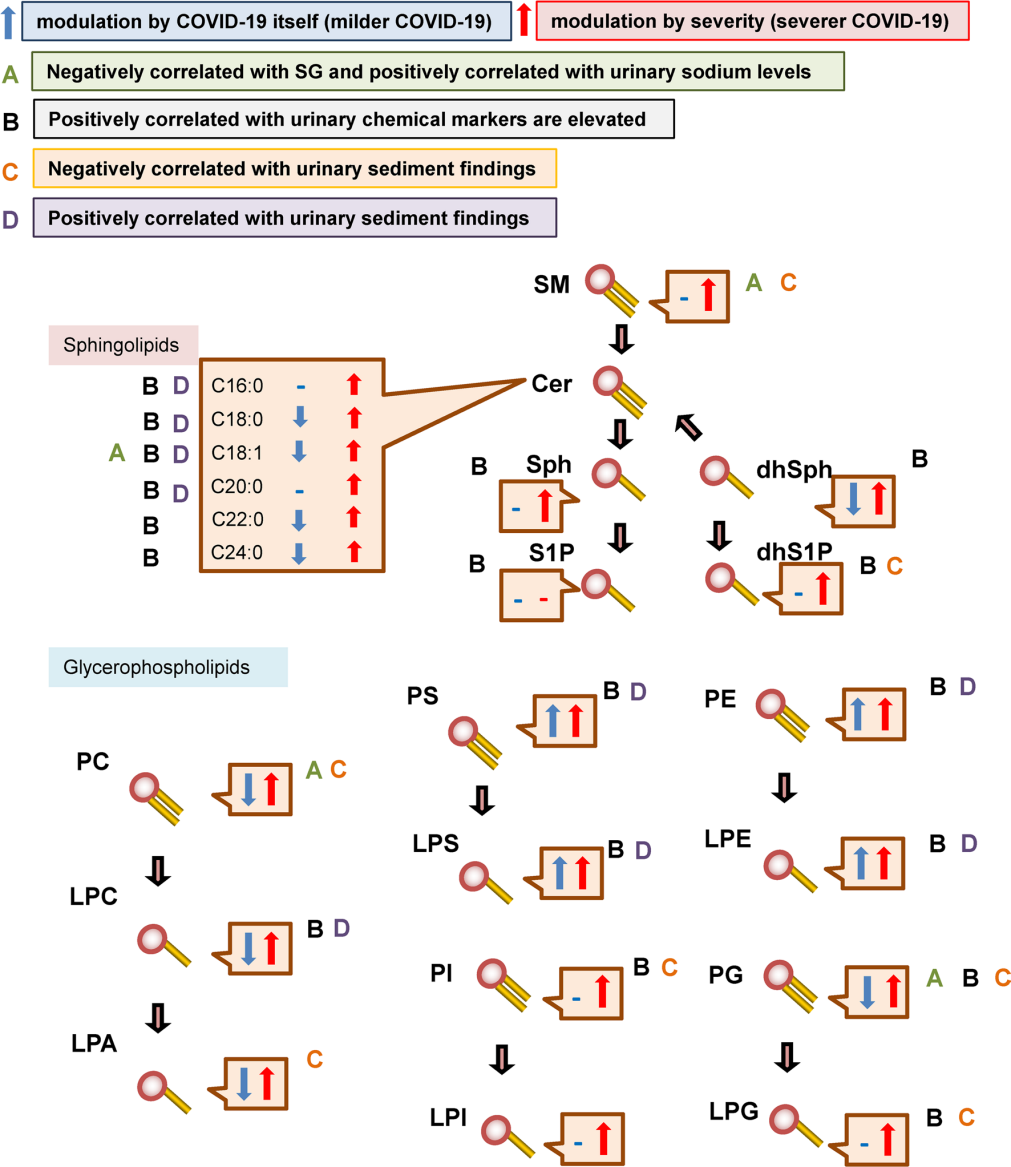
Scheme showing the modulation of urinary sphingolipids and glycerophospholipids in COVID-19. The scheme summarizes the general dynamic modulations of sphingolipids and glycerophospholipids in COVID-19

The present study examined the dynamic modulations of urinary sphingolipids and glycerophospholipids in COVID-19 subjects. The modulations of the monitored lipids are summarized in Fig. 6. The modulations of each representative specie of lipids which were not shown in the previous figures are described in Additional file 1: Figs. S23–S25.

The urinary SM levels increased only in maximum severity group 3 and were positively correlated with the maximum severity of COVID-19, suggesting that SM modulations were not specific to COVID-19-specific factors but were instead related to kidney injuries accompanying severe infection. Contrary to a previous paper reporting that the urinary SM levels were positively correlated with the urinary TP levels [67], the urinary levels of the SM species were not consistently correlated with the urinary TP level. Of note, the urinary total SM level was rather strongly negatively correlated with the urinary SG and positively with sodium levels (Fig. 5). The negative correlations of the SM with the RTE suggested the possibility that the urinary SM levels decrease in response to the prerenal factors, although a previous study reported that sphingomyelinase activities declined in the model of ischemic renal injury [70].

The modulations of urinary ceramides largely depended on the species. Overall, the levels of the monitored ceramides increased, especially in severe COVID-19, with the exception of C18:0 Cer (Fig. 1). Many ceramide species were downregulated during the early phase (day 1–3) in maximum severity group 1, suggesting that the downregulation of ceramides might originate from the direct influence of infection with SARS-CoV-2. A recent report demonstrated that the envelope of SARS-CoV-2 is rich in cholesterol and phospholipids and poor in sphingolipids [54], suggesting that these modulations are unlikely to be explained by the expenditure of sphingolipids during virus replication. During the later phase, the elevation of ceramides in severe COVID-19 might reflect the progression of kidney injuries, since ceramide production was induced in kidney injury models and ceramides are known to induce the apoptosis of renal mesangial cells and renal tubular epithelial cells [3, 18, 32]. Actually, the urinary ceramide levels were positively correlated with urinary chemical biomarkers and the urinary sediment findings in the COVID-19 subjects in the present study (Fig. 5).

The urinary Sph, dhSph, and dhS1P levels increased, especially during the later phase in maximum severity group 3, while they tended to decrease during the early phase (day 1–3) in maximum severity group 1 (Fig. 1). These results suggested that reductions in Sph and dhSph might be direct effects of infection with SARS-CoV-2, while increases in these sphingolipids may occur as responses to kidney injuries associated with COVID-19, as observed for ceramides. Actually, their urinary levels had positive correlations with the urinary chemical markers (Fig. 5). Considering the agonistic properties of dhS1P for S1P receptors and the potential protective properties of S1P receptors against kidney injuries [1, 12, 24, 25], the elevation in urinary dhS1P levels in severe COVID-19 might reflect a compensatory mechanism in response to COVID-19-associated kidney injuries.

Regarding the PC-LPC-LPA axis, the urinary total PC levels increased from the early phase (day 4–6) only in maximum severity group 3, while the total LPA levels increased during the later phase (Fig. 2). However, when the lipid modulations were investigated in detail, many PC, LPC, and LPA species decreased in the COVID-19 subjects (Fig. 3). These results suggested that SARS-CoV-2 infection generally downregulated the PC-LPA-LPA axis in a direct manner, while kidney injuries caused by critical COVID-19 disease resulted in upregulation. Urinary SG levels had rather negative correlations with PC and urinary sodium levels had positive ones with PC, while the urinary PC levels were rather negatively correlated with the RTE, suggesting that the urinary PC levels decrease in response to the prerenal factors. Regarding the pathophysiological significance, since LPA is involved in renal fibrosis as well as inflammation [20, 29, 53] and LPC has been shown to have a strong lipotoxicity in the field of nephrology [69], increases in LPA and LPC, especially during the later phase, might result in the translation of acute kidney injuries into chronic kidney injuries, which has been observed as a sequelae of COVID-19 [43].

Regarding the PS-LPS axis, the urinary PS and LPS levels increased rapidly, especially in patients with severe COVID-19 (Fig. 2). Although modulations of the urinary PS levels in AKI have not been reported, considering that PS is involved in apoptosis [39] and exosome formation [59], the elevation of PS in COVID-19-associated kidney injuries seems reasonable. The urinary PS and LPS levels were positively correlated with urinary chemical biomarkers and urinary sediment findings, suggesting that these levels reflect kidney injuries that have been mainly caused by renal factors. The roles of LPS remain to be elucidated in the fields of nephrology, while we recently demonstrated the elevation of PS-PLA_1_, a producing enzyme for LPS, in the serum of COVID-19 patients [58]. Since LPS and LPS receptors are involved in the regulation of the immune system through three kinds of specific receptors [17, 46], LPS might possess important roles in the pathogenesis of COVID-19-associated kidney injuries, in which inflammation might at least partly be involved [30].

The urinary levels of PE and LPE increased in COVID-19 beginning at an early phase. The upregulation of the PE-LPE axis might be characteristic of COVID-19, as shown in the volcano plots (Fig. 3). PE is abundant in the envelope of SARS-CoV-2 [54] and is reportedly involved in the replication of RNA viruses [66]. Previous studies suggested that LPE might possess anti-inflammatory properties on macrophages [48], which might activate natural killer T cell-dependent protective immunity [71]. Considering these potential biological properties of LPE and the negative correlation between LPE levels during the early phase and maximum severity, LPE might have protective biological properties against the pathogenesis of COVID-19, and a failure to increase LPE levels might be one mechanism resulting in the aggravation of COVID-19.

Regarding the PG-LPG axis and the PI-LPI axis, the roles of PG in AKI are unknown, while PG reportedly suppresses toll-like receptor-mediated inflammation [8], suggesting that a decrease in PG might promote kidney injury. In contrast, the upregulation of LPG in patients with severe COVID-19 might contribute to the acceleration of inflammation, since LPG exerts agonist activities with proinflammatory GPR55 [22, 45]. LPI also acts on GPR55 [45], suggesting that the elevation of LPI during the later phase of severe COVID-19 might accelerate the pathogenesis of COVID-19-associated kidney injuries. Regarding the correlation with clinical phenotypes, the PG-LPG axis and the PI-LPI axis show somehow strange correlations with the urinary chemical markers and the urinary sediment findings (Fig. 5). Some unknown mechanisms are involved for the opposite results in the associations with lipids between the urinary chemical markers and the urinary sediment findings.

To the best of our knowledge, the modulations of sphingolipids and glycerophospholipids in the urine of AKI have not been well studied, while urinary levels of ceramides, SM, LPA, LPC, and PC have been demonstrated to be higher in chronic kidney diseases, especially diabetic nephropathy, as described in the *Introduction* [38, 51, 55, 60, 67, 69]. Although the number of the subjects was limited, when we investigated the association between diabetes and urinary lipid levels in the control subjects used in the present study, we observed that the urinary levels of total LPG and S1P were also higher in the subjects with diabetes as well as LPC and ceramides. These results together with the previous reports suggested that the mechanisms similar to diabetic nephropathy, such as inflammation, oxidative stress, and fibrosis, might be somehow involved in the modulations of sphingolipids and glycerophospholipids in the present study. Anyway, since significant elevation of urinary levels of LPS, PS, LPE, PE, PG, LPI, and PI have not been observed or reported in the urine of chronic kidney diseases, several mechanisms specific to COVID-19 or AKI may be involved in the dynamic modulations of these lipids.

Since this was an observational study, the main limitation is that the possible involvement of these lipid modulations in the pathogenesis of COVID-19-associated kidney injuries remains unknown. However, the accuracy of the models predicting maximum severity that were constructed using machine learning methods was generally high, especially during the early phase of COVID-19 (Fig. 4B and Additional file 1: Fig. S12), and several lipids were selected as important factors, in addition to important clinical parameters that are typically used to predict severity. These results suggest the potential usefulness of these lipids as biomarkers for predicting the maximum severity of COVID-19. Moreover, since the PC-LPA-LPC axis was important throughout the time course, sphingolipids were important during the early phase, and PS and the PE-LPE axis were important during the middle phase in all three machine learning models for predicting maximum severity, these lipids might have some important physiological properties in the pathogenesis of COVID-19 or its associated kidney injuries. Another limitation is that, although we evaluated possible confounding factors affecting the modulations of urinary sphingolipids and glycerophospholipids as described in the first section of the *Results*, we could not completely match the backgrounds of both the COVID-19 and control groups, since some characteristics such as age differed largely among the maximum severity groups. In addition, since the eGFR levels were not obviously modulated in the present study (Additional file 1: Fig. S1H), we were unable to investigate cases with severe AKI. At present, we could not conclude whether the urinary modulations of the lipids would recover when the patients are cured of COVID-19. Although the data is preliminary since the number of samples were limited, the urinary lipid levels generally recovered to the range of control subjects and no obvious differences among the maximum severity were observed except that the total PE levels were still higher in the subjects who had recovered from severe COVID-19 (Additional file 1: Fig. S26). Considering that serum modulation of lipids maintained for a long time [4, 65], further studies with post-COVID-19 subjects are necessary in the future to elucidate the mechanisms for long COVID-19.

## Conclusions

In summary, analyses of urine samples collected from COVID-19 subjects showed that decreases in the urinary levels of C18:0, C18:1, C22:0, and C24:0 ceramides, Sph, dhSph, PC, LPC, LPA, and PG and increases in those of PS, LPS, PE, and LPE, especially during the early phase, might be derived from the direct effects of SARS-CoV-2 infection, while increases in the urinary levels of SM, ceramides, Sph, dhSph, dhS1P, PC, LPA, PS, LPS, PE, LPE, PG, LPG, PI, and LPI, especially during the later phase, might result from kidney injuries accompanying severe COVID-19. We believe that these results may prompt researchers to perform further investigations to develop laboratory testing methods based on sphingolipid and glycerophospholipid modulations for predicting the maximum severity of COVID-19 and/or novel reagents to suppress the renal complications of COVID-19.

## Supplementary Information

**Additional file 1**. Supplementary tables and figures.

## Data Availability

All the data was included in the manuscript and Additional file 1. The datasets generated or analyzed in the current study will be made available upon reasonable request. All materials are commercially available.

## Abbreviations

AKI: Acute kidney injury
ACE2: Angiotensin-converting enzyme 2
α1-MG: α1-Microglobulin
COVID-19: Coronavirus disease 2019
C16:0 Cer: D18:1/16:0 ceramide
C18:0 Cer: D18:1/18:0 ceramide
C18:1 Cer: D18:1/18:1 ceramide
C20:0 Cer: D18:1/20:0 ceramide
C22:0 Cer: D18:1/22:0 ceramide
C24:0 Cer: D18:1/24:0 Ceramide
dhS1P: Dihydrosphingosine 1-phosphate
dhSph: Dihydrosphingosine
GraC: Granular casts
HPF: High-power field of view
HyaC: Hyaline casts
L-FABP: Liver type fatty acid-binding protein
LPA: Lysophosphatidic acid
LPC: Lysophosphatidylcholine
LPE: Lysophosphatidylethanolamine
LPG: Lysophosphatidylglycerol
LPI: Lysophosphatidylinositol
LPS: Lysophosphatidylserine
NAG: *N*-acetyl-β-d-glucosaminidase
NGAL: Neutrophil gelatinase-associated lipocalin
PC: Phosphatidylcholine
PE: Phosphatidylethanolamine
PI: Phosphatidylinositol
PG: Phosphatidylglycerol
PS: Phosphatidylserine
RTE: Renal tubular epithelial cells
RTEC: Epithelial casts
S1P: Sphingosine 1-phosphate
SARS-CoV-2: Severe acute respiratory syndrome coronavirus 2
SM: Sphingomyelin
Sph: Sphingosine
TP: Urinary total protein
WaxC: Waxy casts
WF: Whole field
