# Supplementary material for: Dynamic modulations of urinary sphingolipid and glycerophospholipid levels in COVID-19 and correlations with COVID-19-associated kidney injuries
Source: J Biomed Sci. 2022 Nov 10;29:94. doi: 10.1186/s12929-022-00880-5 (PMC9647768; doi:10.1186/s12929-022-00880-5)
Supplement: Supplementary file 1 — Additional file 1. Supplementary tables and figures. [file 12929_2022_880_MOESM1_ESM.pdf]

Supplemental Table S1. Characteristics of patients

	Control	All COVID-19	Maximum severity 1	Maximum severity 2	Maximum severity 3	*p* value
Number of subjects	95.0	91.0	25.0	45.0	21.0	
Age	61.2 ± 12.5	63.9 ± 15.4	54.4 ± 17.8	67.2 ± 14.5	68.0 ± 9.2	1 vs. 2*, 1 vs. 3*
Sex (male%)	72.6	69.9	64.0	64.4	76.2	N.S.
Diabetes (%)	46.3	43.5	32.0	44.4	42.9	N.S.
Hypertension (%)	36.8	46.8	40.0	66.7	57.1	$p = .006$
Current smoking (%)	18.9	16.7	4.0	13.3	28.6	N.S.

The difference in age was evaluated using the Kruskal-Wallis test, followed by the Steel-Dwass test as a post-hoc test. For the difference in sex and the presence of diabetes, hypertension, and current smoking, χ^2 square analyses were performed.

Supplemental Tables S2. Characteristics of patients from whom samples were collected at a specific time point

A. Day 1–3

Maximum Severity	1	2	3	Statistical description
Number of samples	7	2		
Number of patients	7	2		
Age	55.3 ± 20.7	67.5 ± 11.5	±	
Sex (male%)	57.1	100.0		
Diabetes (%)	14.3	50.0		
Hypertension (%)	14.3	100.0		
Current smoking (%)	0.0	0.0		

B. Day 4–6

Maximum Severity	1	2	3	Statistical description
Number of samples	5	13	6	
Number of patients	5	8	5	
Age	51.0 ± 14.0	77.2 ± 14.1*	77.5 ± 5.9*	1 vs. 2*, 1 vs. 3*
Sex (male%)	80.0	38.5	50.0	
Diabetes (%)	40.0	30.8	50.0	
Hypertension (%)	60.0	84.6	33.3	p = .029
Current smoking (%)	0.0	15.4	33.3	

C. Day 7–9

Maximum Severity	1	2	3	Statistical description
Number of samples	10	28	10	
Number of patients	10	18	6	
Age	54.4 ± 17.9	67.0 ± 12.3	74.5 ± 4.5*	1 vs. 3*
Sex (male%)	50.0	64.3	70.0	
Diabetes (%)	40.0	53.6	40.0	
Hypertension (%)	50.0	67.9	30.0	
Current smoking (%)	10.0	7.1	40.0	

D. Day 10–12

Maximum Severity	1	2	3	Statistical description

Number of samples	7	19	23	
Number of patients	6	15	13	
Age	60.9 ± 11.1	59.7 ± 14.3	68.3 ± 7.1	
Sex (male%)	85.7	73.7	91.3	
Diabetes (%)	42.9	63.2	39.1	
Hypertension (%)	57.1	57.9	73.9	$p = .004$
Current smoking (%)	0.0	15.8	30.4	

E. Day 13–15

Maximum Severity	1	2	3	Statistical description
Number of samples	3	30	28	
Number of patients	2	19	16	
Age	56.3 ± 14.6	64.8 ± 11.4	68.2 ± 10.0	
Sex (male%)	66.7	83.3	82.1	
Diabetes (%)	33.3	60.0	50.0	
Hypertension (%)	100.0	73.3	75.0	$p < .001$
Current smoking (%)	0.0	13.3	17.9	

F. Day 16–18

Maximum Severity	1	2	3	Statistical description
Number of samples		13	22	
Number of patients		10	12	
Age		67.4 ± 7.4	68.2 ± 10.0	
Sex (male%)		84.6	100.0	
Diabetes (%)		53.8	50.0	
Hypertension (%)		76.9	63.6	
Current smoking (%)		7.7	31.8	

G. Day 19–24

Maximum Severity	1	2	3	Statistical description
Number of samples		4	11	
Number of patients		3	7	
Age		71.0 ± 8.7	62.5 ± 11.3	
Sex (male%)		100.0	90.9	$p = .016$
Diabetes (%)		50.0	63.6	
Hypertension (%)		75.0	72.7	$p = .004$
Current smoking (%)		0.0	27.3	

H. Day 25–40

Maximum Severity	1	2	3	Statistical description
Number of samples	1	6	34	
Number of patients	1	5	11	
Age	54	66.7 ± 15.0	60.2 ± 8.6	
Sex (male%)	100.0	83.3	97.1	$p = .029$
Diabetes (%)	100.0	33.3	61.8	
Hypertension (%)	0.0	50.0	94.1	$p < .001$
Current smoking (%)	0.0	16.7	26.5	

The difference in age was evaluated using the Kruskal-Wallis test, followed by the Steel-Dwass test as a post-hoc test. For the difference in sex and the presence of diabetes, hypertension, and current smoking, χ^2 square analyses were performed.

Supplemental Tables S3. Accuracy of the machine learning models predicting maximum severity in independent urine samples

	CHAID	SVM	neural network
day 1-6	100.0	85.7	71.4
day 7-9	90.0	80.0	90.0
day 10-12	80.0	90.0	80.0
day 13-15	66.7	83.3	83.3
day 16-18	100.0	75.0	100.0
day 19-40	100.0	87.5	100.0
All	89.1	84.8	89.1

The accuracy (%) of the machine learning models constructed in Figure 4B and Supplemental Figure S12 was examined with independent urine samples used for the validation of the results.

Supplemental Figure S1

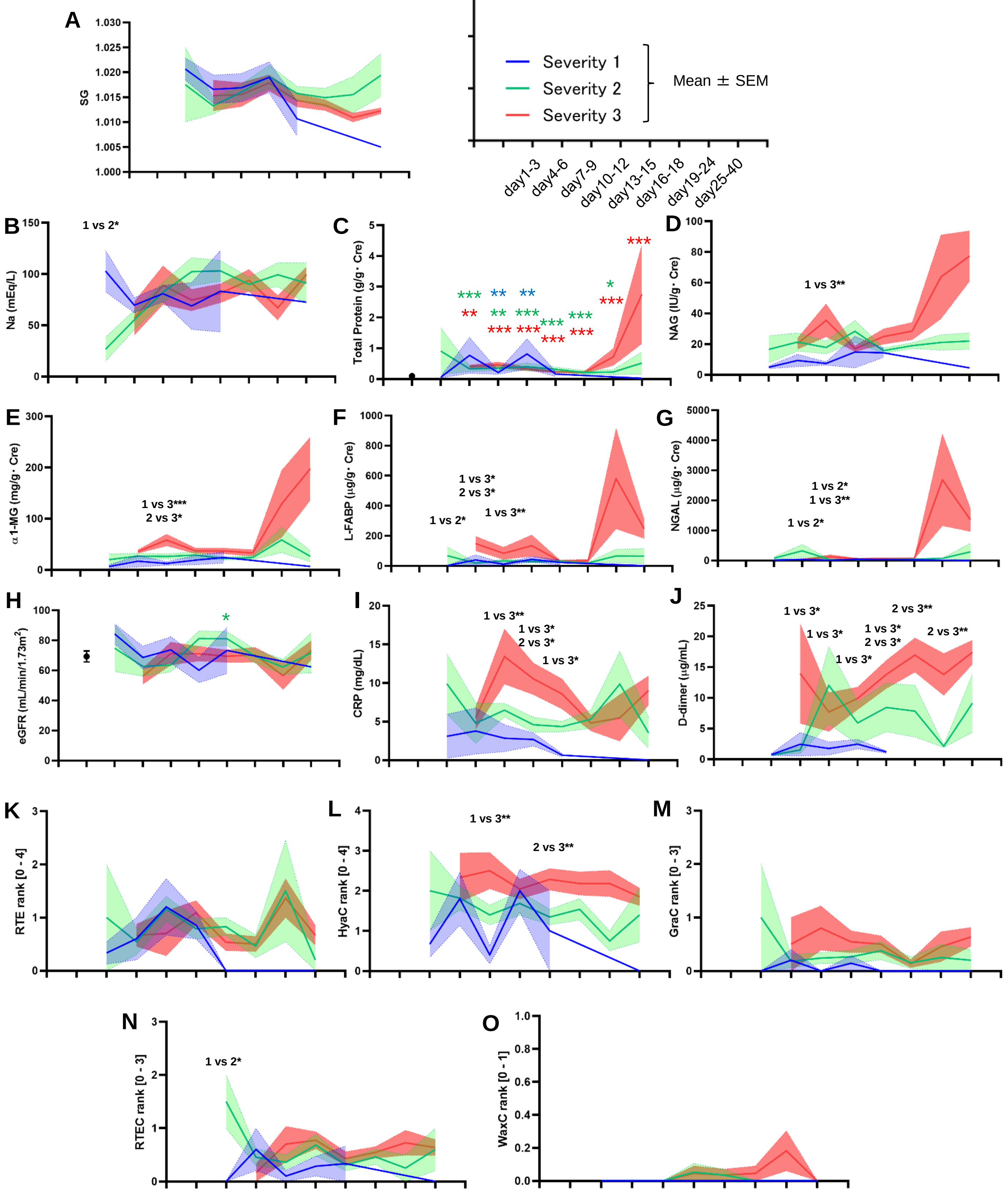

Supplemental Figure S1. Time courses of clinical parameters. The time courses of the clinical parameters are shown in a manner similar to that used in Figures 1 and 2.

Supplemental Figure S2

Severity 2

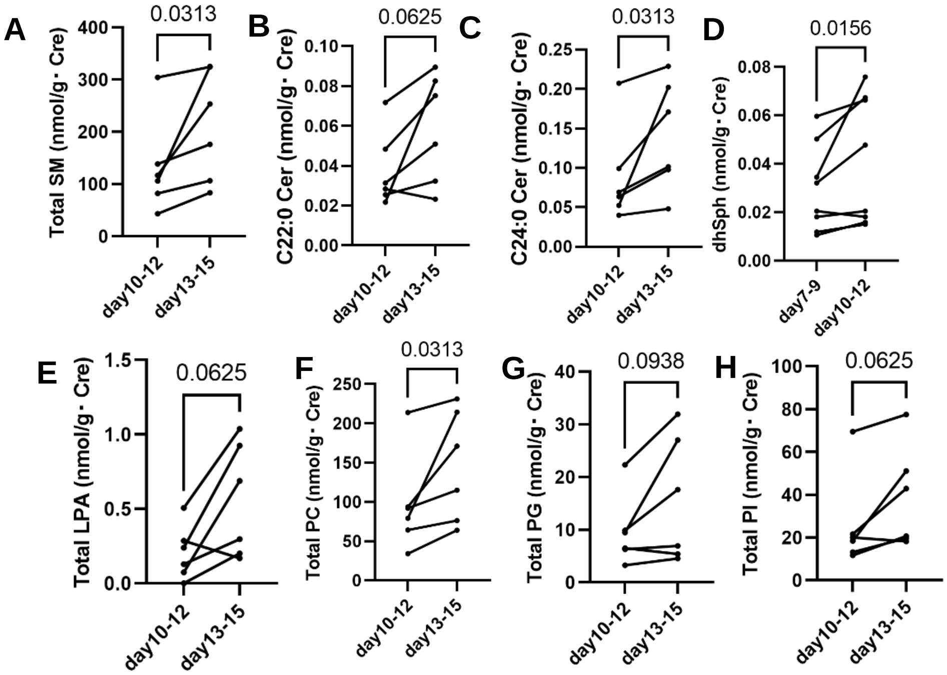

Supplemental Figure S2. Paired comparison of the urinary lipid levels between specific time points in maximum severity group 2. The difference in the urinary lipid levels between specific time points was longitudinally evaluated with the paired Wilcoxon signed-rank test in maximum severity group 2. The differences with *p* <.10 were shown.

Supplemental Figure S3

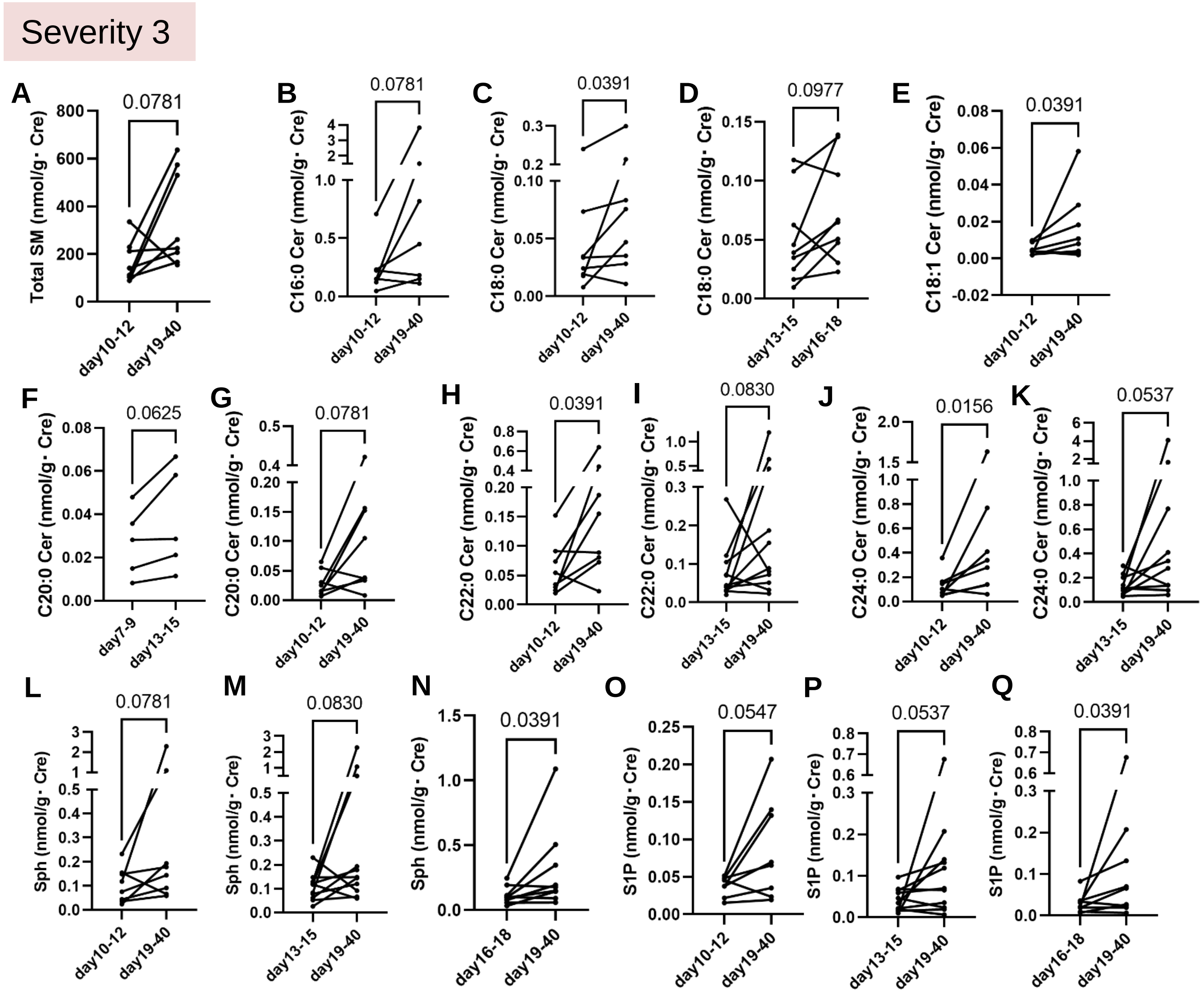

Supplemental Figure S3. Paired comparison of the urinary sphingolipid levels between specific time points in maximum severity group 3. The difference in the urinary sphingolipid levels between specific time points was longitudinally evaluated with the paired Wilcoxon signed-rank test in maximum severity group 3. The differences with *p* <.10 were shown.

Supplemental Figure S4

Severity 3

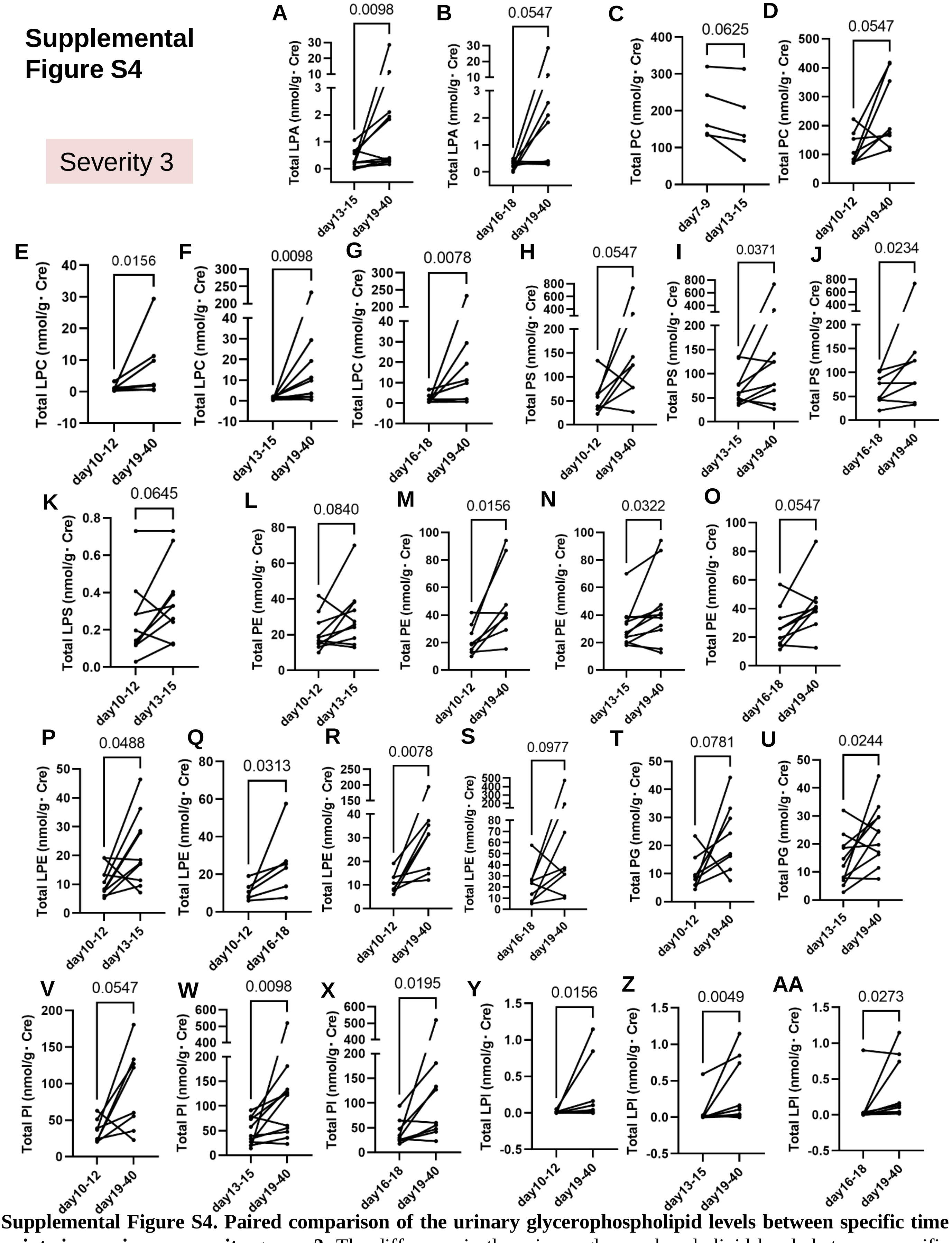

Supplemental Figure S4. Paired comparison of the urinary glycerophospholipid levels between specific time points in maximum severity group 3. The difference in the urinary glycerophospholipid levels between specific time points was longitudinally evaluated with the paired Wilcoxon signed-rank test in maximum severity group 3. The differences with $p < .10$ were shown.

Supplemental Figure S5

day 1 – 3

A

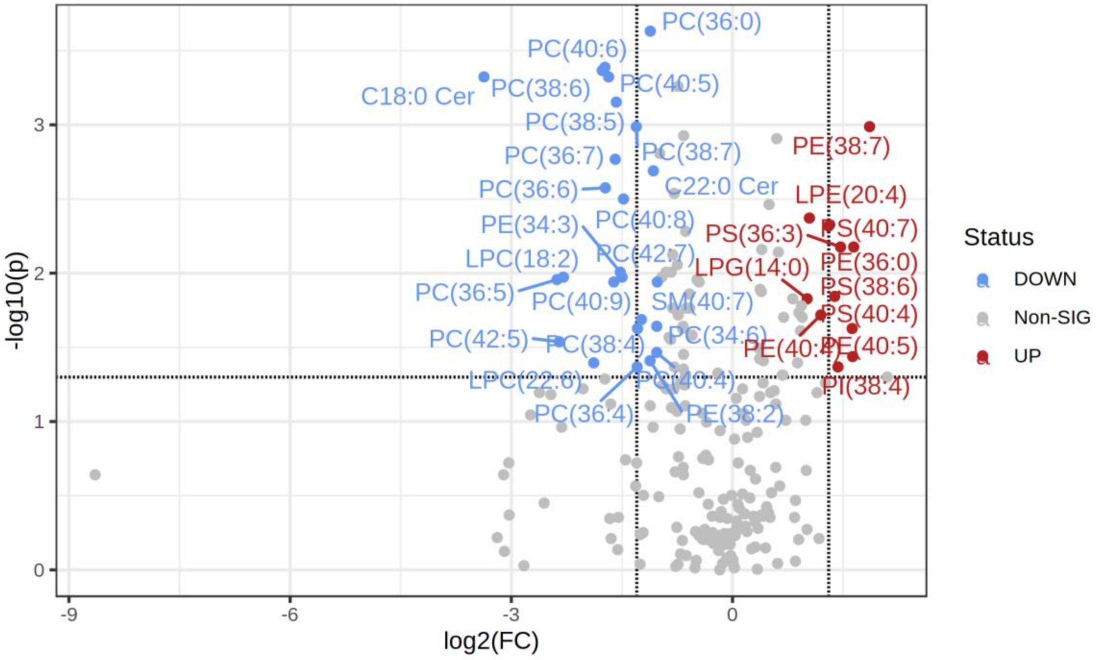

day 4 – 6

B

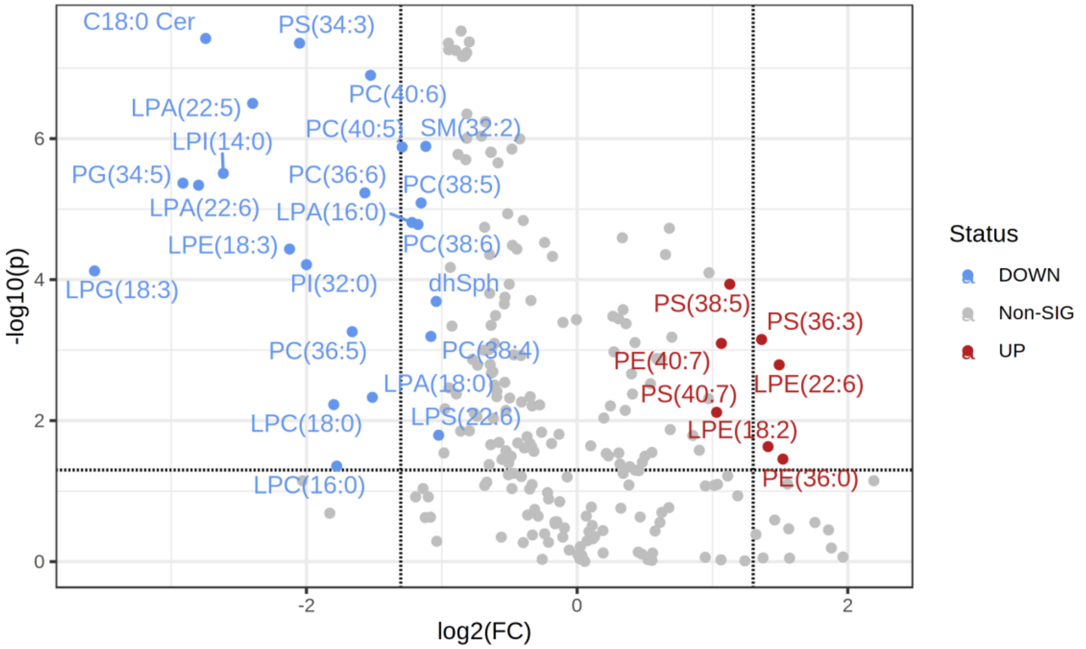

Control → **COVID-19**

Supplemental Figure S5. Volcano plots for identifying significant lipids capable of differentiating COVID-19 on day 1–3 and day 4–6. Volcano plots were used to investigate significant lipids capable of differentiating COVID-19 subjects from control subjects on day 1–3 (A) and day 4–6 (B).

Supplemental Figure S6

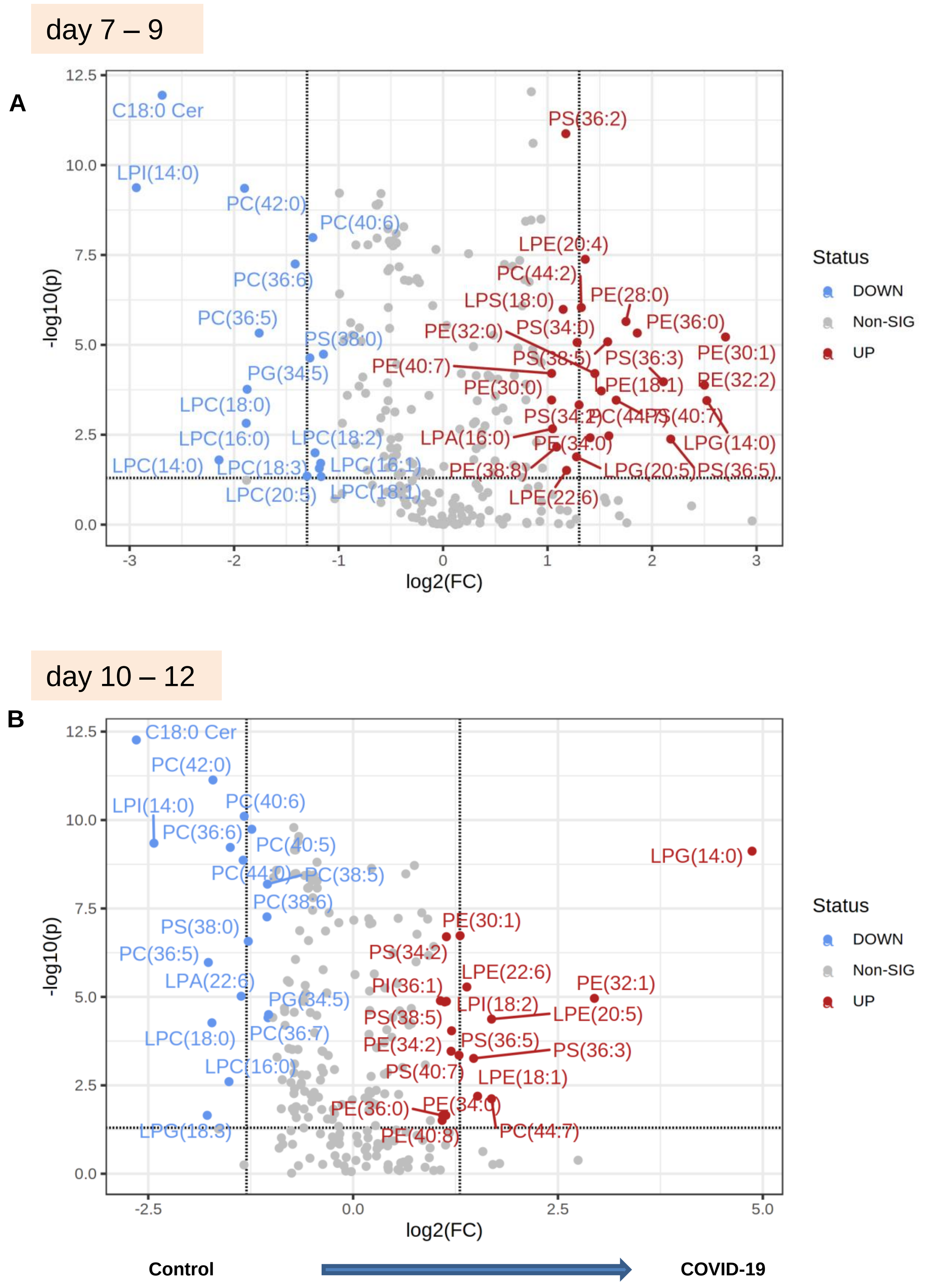

Supplemental Figure S6. Volcano plots for identifying significant lipids capable of differentiating COVID-19 on day 7–9 and day 10–12. Volcano plots were used to investigate significant lipids capable of differentiating COVID-19 subjects from control subjects on day 7–9 (A) and day 10–12 (B).

Supplemental Figure S7

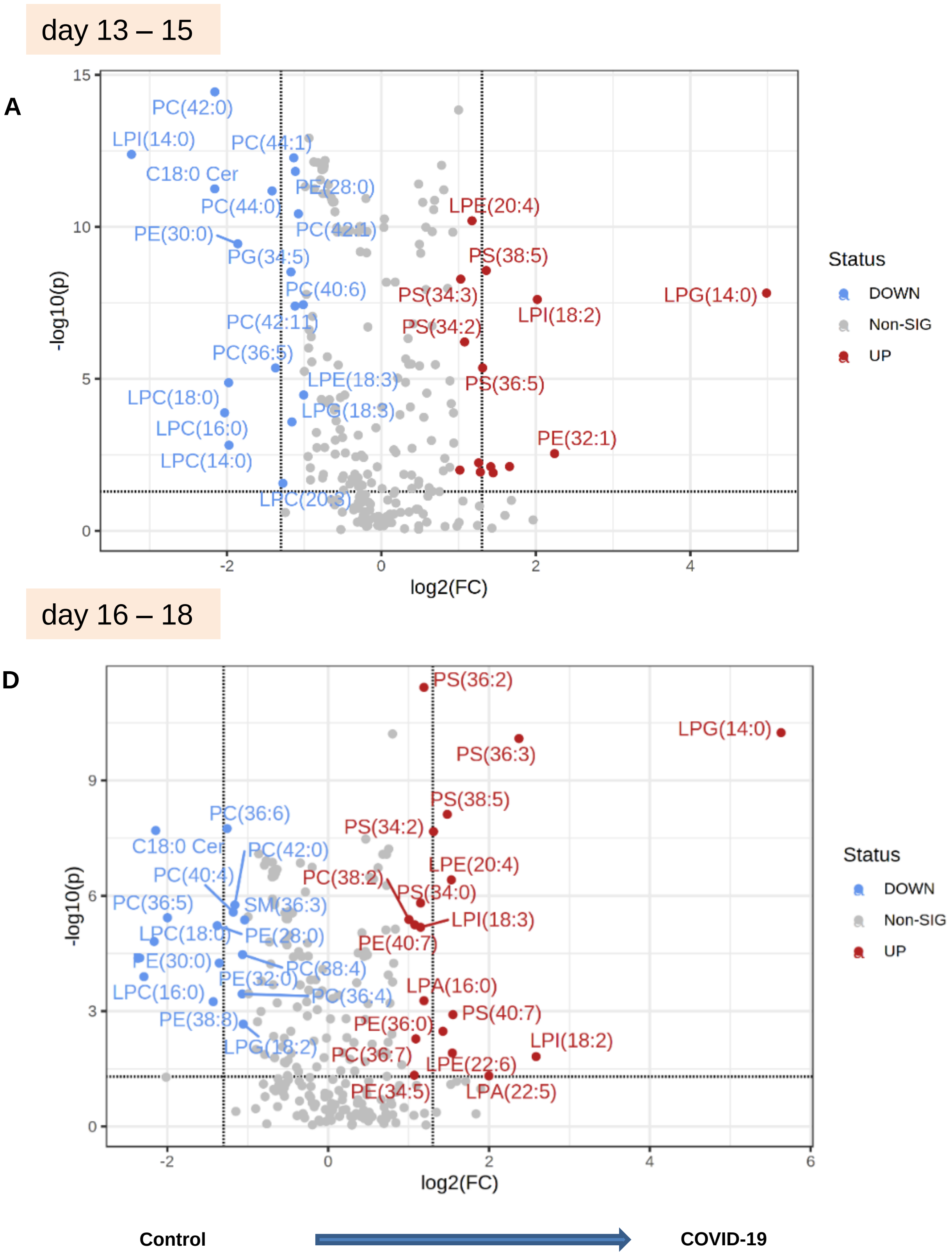

Supplemental Figure S7. Volcano plots for identifying significant lipids capable of differentiating COVID-19 on day 13–15 and day 16–18. Volcano plots were used to investigate significant lipids capable of differentiating COVID-19 subjects from control subjects on day 13–15 (A) and day 16–18 (B).

Supplemental Figure S8

day 19 – 24

A

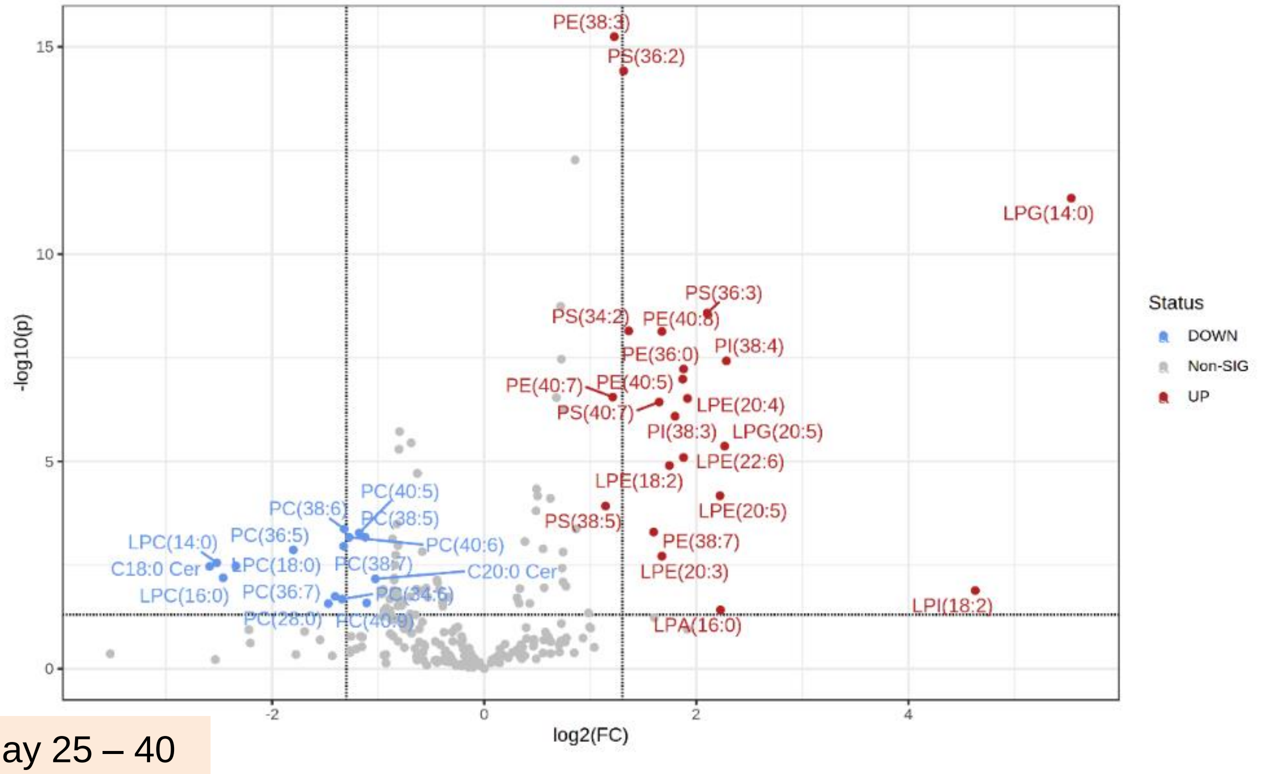

day 25 – 40

B

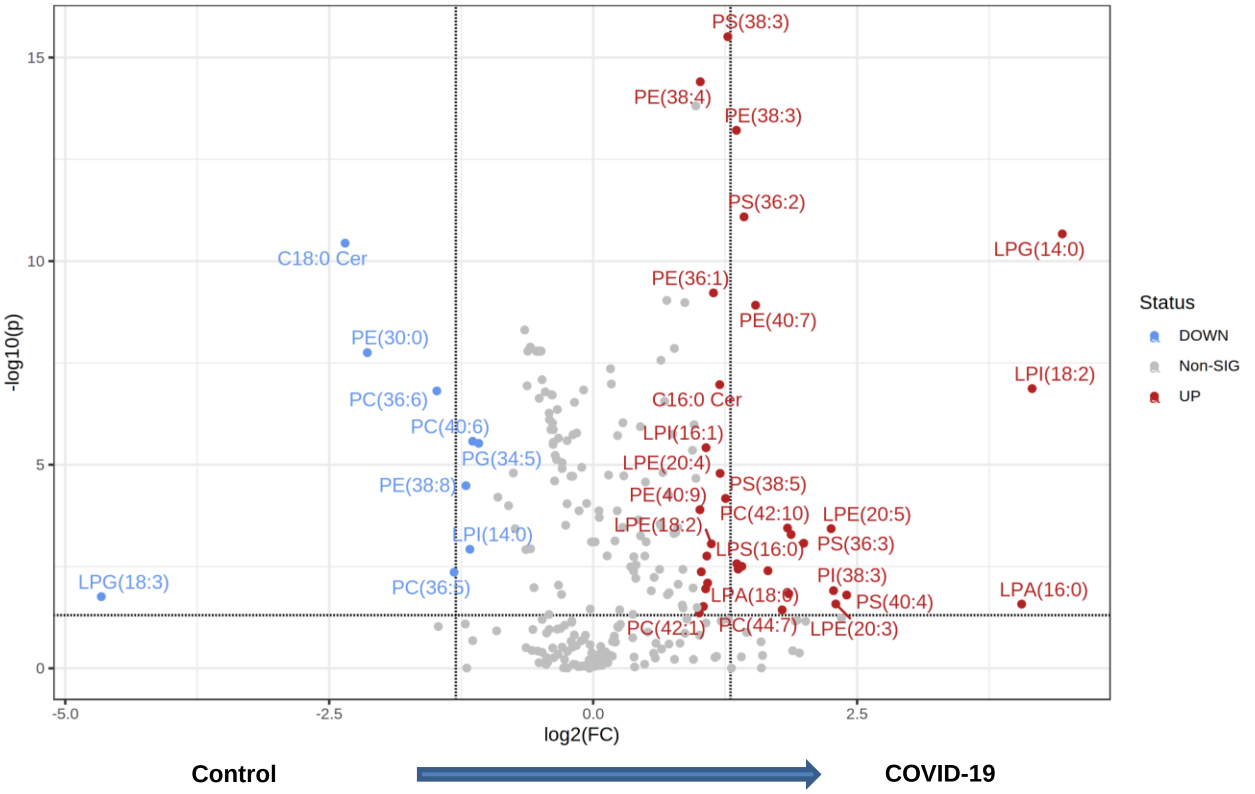

Supplemental Figure S8. Volcano plots for identifying significant lipids capable of differentiating COVID-19 on day 19–24 and day 25–40. Volcano plots were used to investigate significant lipids capable of differentiating COVID-19 subjects from control subjects on day 19–24 (A) and day 25–40 (B).

Supplemental Figure S9

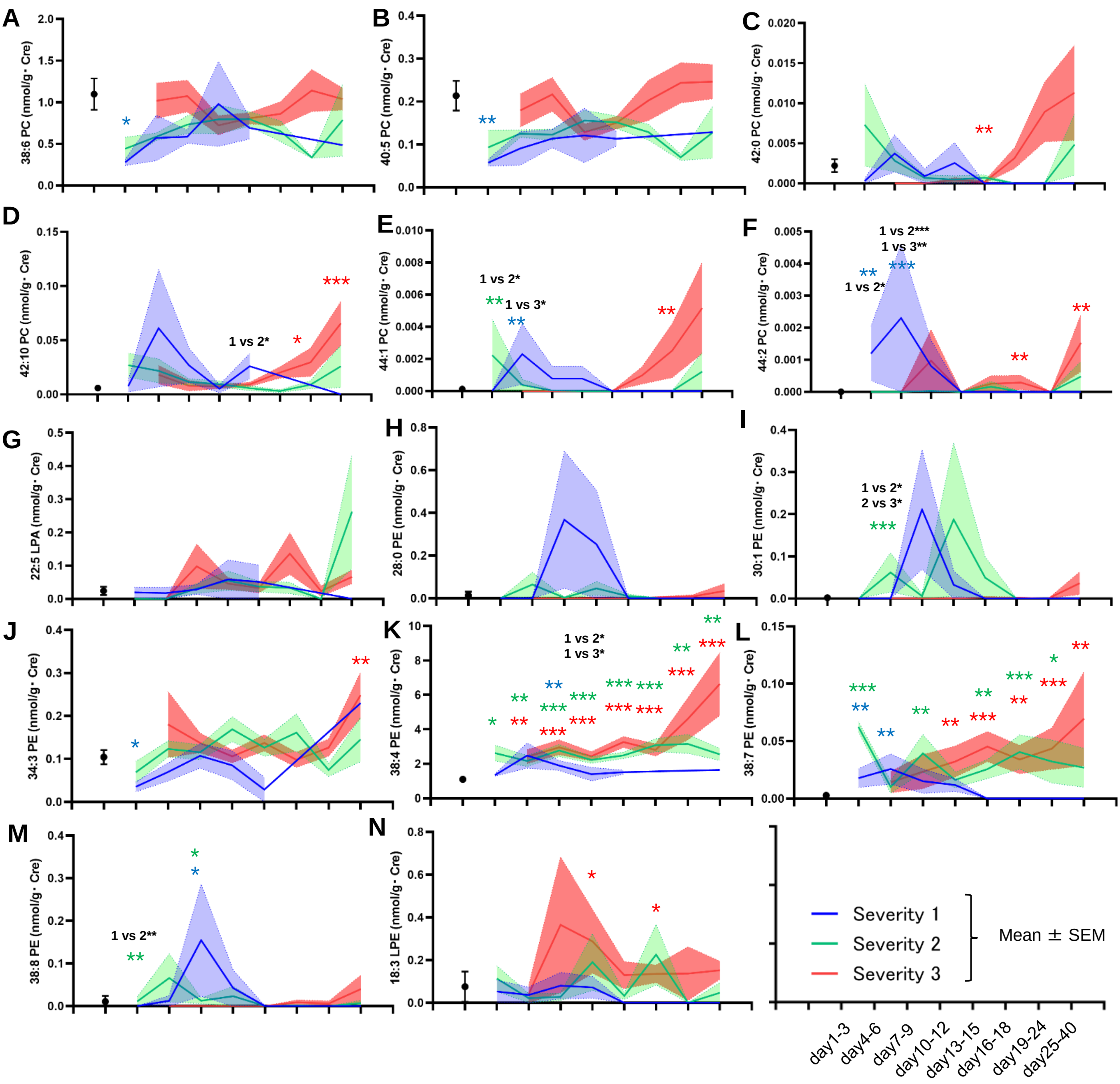

Supplemental Figure S9. Time courses of representative lipids in Figure 3A. The time courses of representative lipids in Figure 3A are shown in a manner similar to that used in Figures 1 and 2.

Supplemental Figure S10

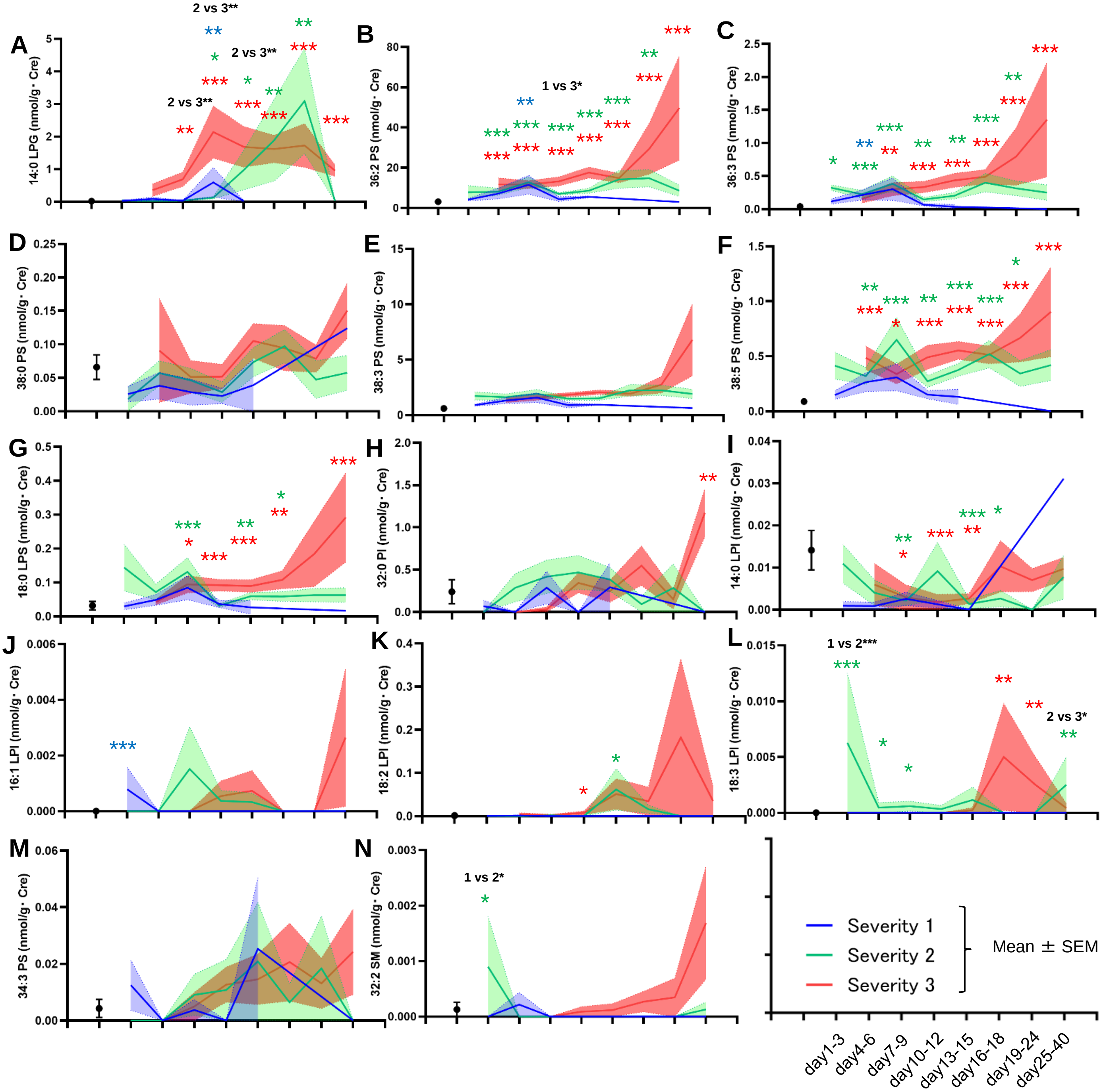

Supplemental Figure S10. Time courses of representative lipids in Figure 3A (continued). The time courses of representative lipids in Figure 3A are shown in a manner similar to that used in Figures 1 and 2.

Supplemental Figure S11

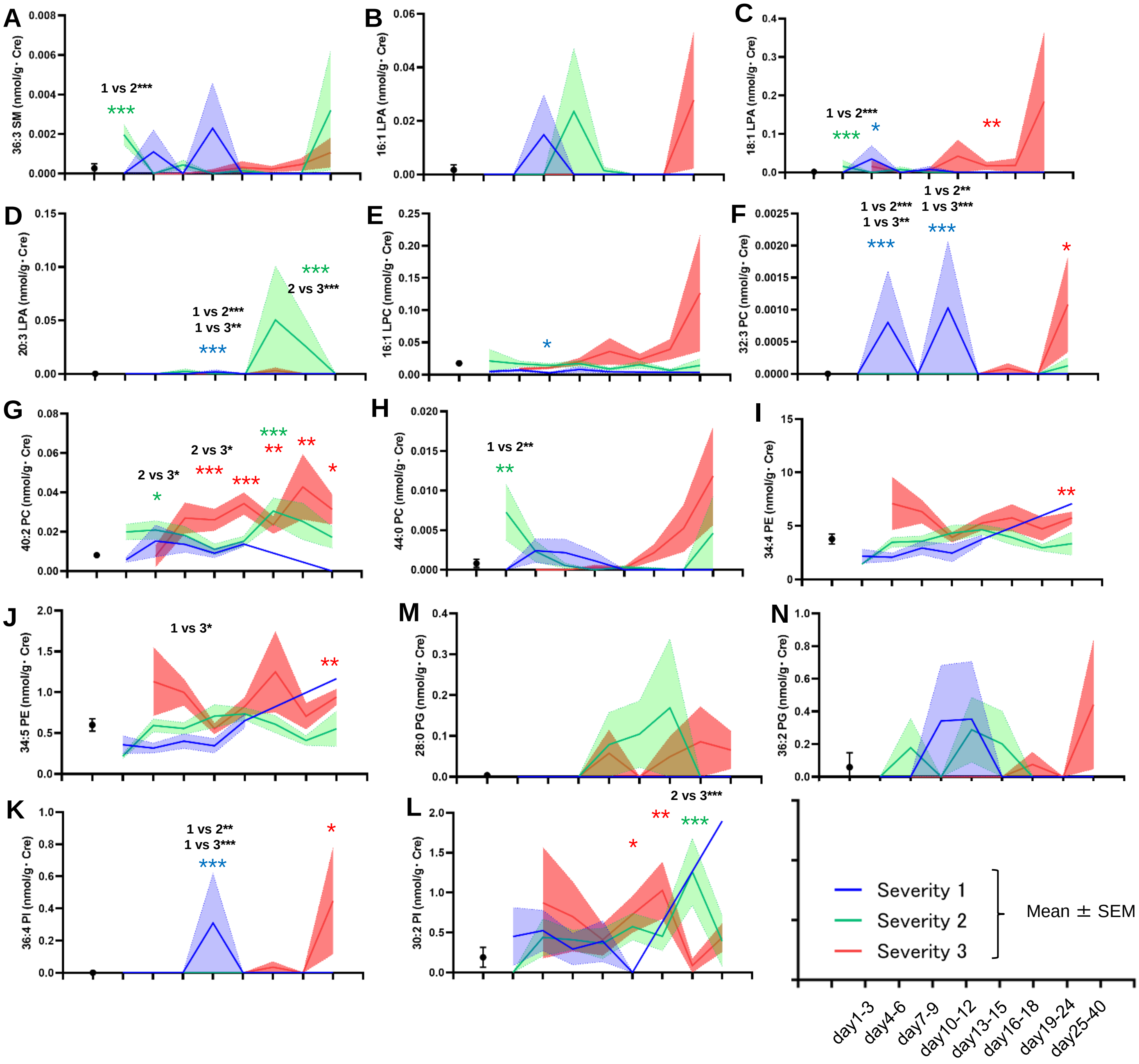

Supplemental Figure S11. Time courses of representative lipids in Figure 4. The time courses of representative lipids in Figure 4 are shown in a manner similar to that used in Figures 1 and 2.

Supplemental Figure S12

A

day 1-6 (96.6 %)		day7-9 (93.3 %)		day 10-12 (91.7 %)		day13-15 (85.0 %)		day16-18 (97.1%)		day19-40 (92.7%)	
parameter	importance	parameter	importance	parameter	importance	parameter	importance	parameter	importance	parameter	importance
SM(38:4)	0.031	SM(38:3)	0.0615	PC(34:4)	0.059	HyaCrank	0.1346	HyaCrank	0.1013	D-Dimer	0.1321
PC(34:5)	0.0289	PC(34:5)	0.0609	SM(38:3)	0.0548	CRP	0.0304	smoking	0.0816	PS(34:4)	0.0636
PC(34:4)	0.0287	PC(34:4)	0.0565	SM(38:4)	0.0532	PS(36:2)	0.028	LPE(20:5)	0.06	PS(28:0)	0.0624
U-Na	0.0265	GraCrank	0.0475	LPA(18:0)	0.0518	dhS1P	0.0206	PC(42:5)	0.06	PC(34:1)	0.0563
SM(38:3)	0.0252	SM(38:4)	0.039	PC(34:5)	0.0488	PS(38:4)	0.0197	PC(36:6)	0.06	PC(34:2)	0.0562
D-Dimer	0.0191	RTErank	0.0371	PS(38:5)	0.0298	PS(38:5)	0.0188	PC(42:0)	0.06	PC(36:4)	0.056
SM(38:5)	0.0176	PE(30:0)	0.037	PS(36:2)	0.0252	Alb/Cr	0.0176	PC(44:12)	0.06	PC(34:6)	0.056
HyaCrank	0.0176	CRP	0.0305	age	0.0227	PS(34:1)	0.0152	PC(42:11)	0.06	PC(36:0)	0.0559
HT	0.016	HyaCrank	0.0283	SM(38:5)	0.0225	αMG/Cr	0.0151	PC(40:10)	0.06	PC(40:5)	0.0559
LPA(22:5)	0.0132	αMG/Cr	0.0146	PS(36:3)	0.0224	PS(36:3)	0.0144	PC(42:10)	0.06	PG(32:0)	0.0559
αMG/Cr	0.0124	SM(38:5)	0.0128	PS(40:7)	0.0214	PS(40:4)	0.0142	PC(32:0)	0.06	PC(42:2)	0.0559
age	0.0124	LPA(22:6)	0.012	PS(38:4)	0.021	PS(34:2)	0.014	dhSph	0.0599	PC(38:4)	0.0559
eGFR	0.0117	age	0.0118	PS(34:1)	0.0204	PS(38:3)	0.0133	PC(36:2)	0.0599	PC(36:2)	0.0559
C16:0 Cer	0.0116	NAG/Cr	0.011	PS(38:3)	0.0164	SM(34:1)	0.0133	PS(40:7)	0.0577	PC(36:3)	0.0559
PS(38:5)	0.0114	PS(34:5)	0.0097	smoking	0.0121	PC(30:1)	0.013	PS(36:4)	0.0573	PC(38:5)	0.0559

importance

B

day 1-6 (93.1 %)		day7-9 (88.9 %)		day 10-12 (89.6 %)		day13-15 (95.0 %)		day16-18 (100 %)		day19-40 (89.1 %)	
parameter	importance	parameter	importance	parameter	importance	parameter	importance	parameter	importance	parameter	importance
U-Na	0.0335	age	0.038	PS(28:0)	0.0363	LPC(18:0)	0.0374	PS(36:4)	0.1209	PS(34:4)	0.0998
PC(42:7)	0.0275	LPG(14:0)	0.0283	PS(36:0)	0.0352	LPG(22:6)	0.0323	D-Dimer	0.0968	LPA(20:3)	0.0957
D-Dimer	0.0253	dhSph	0.0263	PC(36:0)	0.0302	LPC(16:0)	0.0314	LPE(20:5)	0.0964	PC(36:4)	0.0945
PC(38:0)	0.0232	PE(30:1)	0.0236	SM(32:1)	0.0294	CRP	0.0307	PC(32:0)	0.0912	PC(40:5)	0.0936
SM(36:1)	0.0221	PC(38:4)	0.0226	C24:0 Cer	0.0286	PS(40:4)	0.0307	PC(42:10)	0.0882	D-Dimer	0.0874
L-FABP/Cr	0.0219	PC(36:3)	0.0214	CRP	0.0255	Alb/Cr	0.0304	PC(40:10)	0.0829	HyaCrank	0.0664
LPG(14:0)	0.0207	PC(34:5)	0.0202	SM(40:1)	0.0254	SM(36:2)	0.0297	dhSph	0.0763	PG(32:0)	0.066
PS(38:1)	0.0201	C18:0 Cer	0.0201	LPA(18:0)	0.0244	PC(36:1)	0.0289	PC(36:6)	0.063	PC(36:3)	0.0648
PC(30:1)	0.0193	PE(30:0)	0.0198	LPG(14:0)	0.022	PC(40:9)	0.0278	PC(44:12)	0.0536	PC(36:0)	0.0643
αMG/Cr	0.0189	PE(32:2)	0.0198	PS(38:3)	0.0218	PE(36:4)	0.0268	PC(42:0)	0.0517	PS(28:0)	0.0617
PC(38:1)	0.018	LPC(18:1)	0.0191	PS(38:5)	0.0215	SM(38:1)	0.0261	PC(42:5)	0.0466	PC(38:5)	0.0406
SM(36:2)	0.0177	LPC(18:0)	0.0186	PS(38:4)	0.0212	αMG/Cr	0.0241	smoking	0.0413	PC(34:6)	0.0365
SM(40:2)	0.0176	PC(38:2)	0.0183	PS(40:5)	0.021	PE(38:3)	0.0239	PS(40:7)	0.038	PC(42:2)	0.034
SM(40:1)	0.0167	SM(38:6)	0.018	PS(36:1)	0.0201	PC(38:3)	0.0234	PC(42:11)	0.0293	PC(36:2)	0.0325
PC(40:8)	0.0165	PS(34:4)	0.0178	PC(32:0)	0.0201	PC(38:2)	0.023	PC(36:2)	0.0136	PC(38:4)	0.03

importance

Supplemental Figure S12. Lipids or clinical parameters selected as having a high importance in machine learning analyses. The lipids or clinical parameters selected as having high importance by SVM analyses (A) and neural network analyses (B) were used to construct a machine learning model capable of predicting the maximum severity of COVID-19 using data collected at specific time points.

Supplemental Figure S13

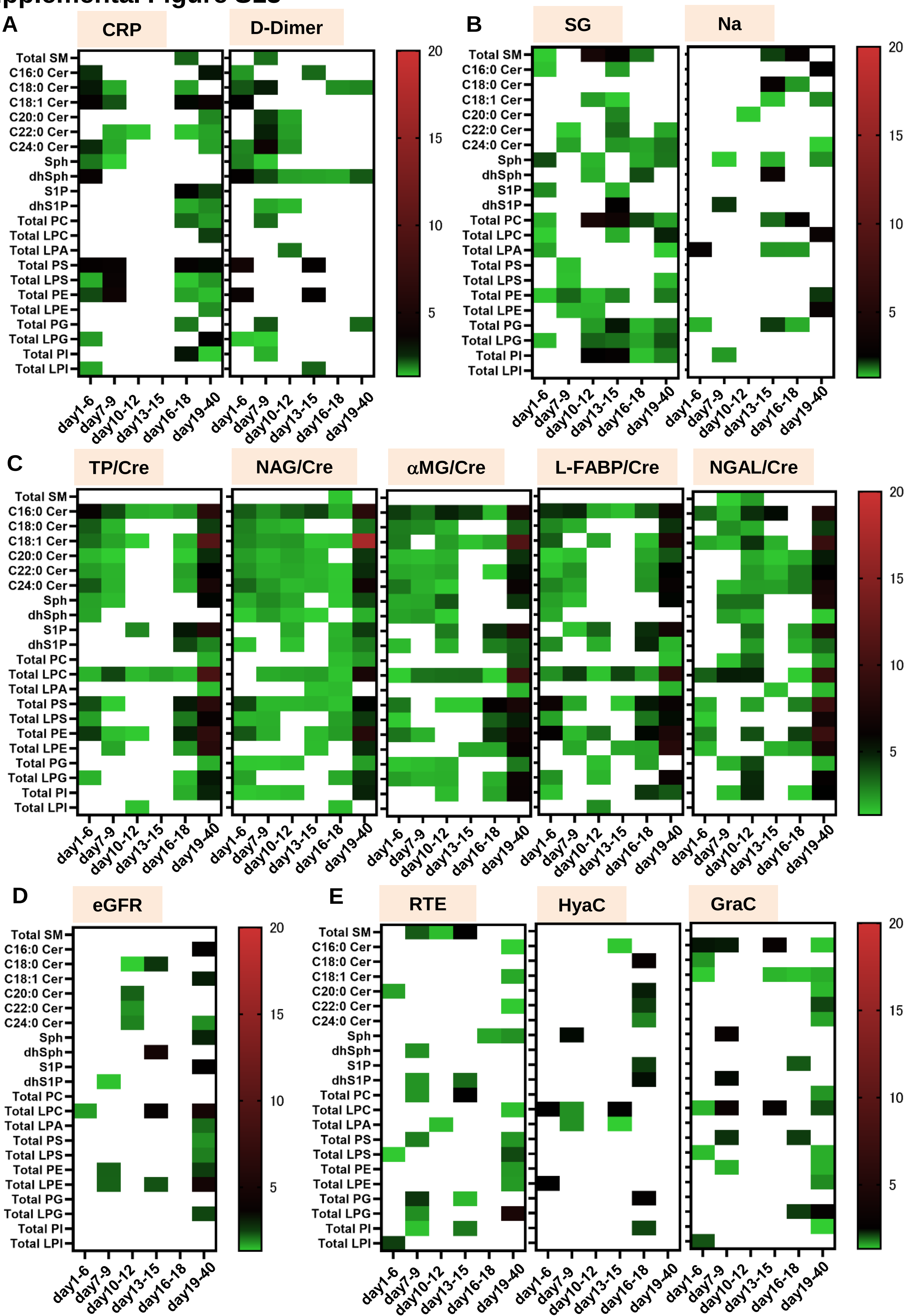

Supplemental Figure S13. Correlations of monitored lipids with clinical parameters. *P* values (shown as $-\log_{10}$ [*p* value]) corresponding to Figure 5 are shown as a heat map.

Supplemental Figure S14

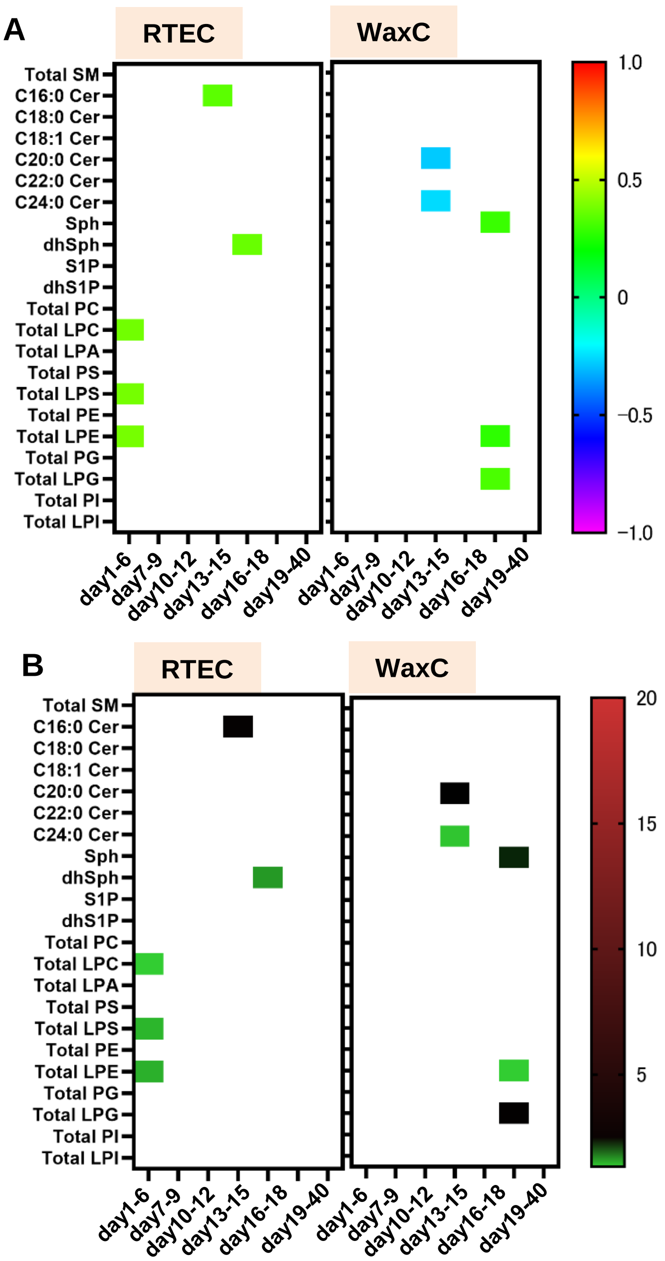

Supplemental Figure S14. Correlations of monitored lipids with the rank of RTEC and WaxC. Spearman rank correlation analyses were used to compare clinical parameters the rank of RTEC and WaxC. (A) Correlation coefficients. (B) *P* values (shown as -$\log_{10}$ [*p* value]). The correlation coefficients and *p* values of non-significant results are shown as blanks.

Supplemental Figure S15

A

	Total SM			S1P			dhS1P		
	variables	β	P value	variables	β	P value	variables	β	P value
day1-6	D-Dimer	1.070	1.745E-11	D-Dimer	1.300	1.336E-10	L-FABP/Cr	0.790	1.557E-06
	pH	0.340	0.0002843	NGAL/Cr	0.538	2.498E-05			
	DM	-0.357	0.000195	smoking	-0.562	0.0004597			
	sex	0.188	0.0215951						
day7-9	max severity	0.524	0.0005276				U-Na	0.484	0.0045989
	RTECrank	-0.327	0.0162694				eGFR	0.340	0.0382208
	NAG/Cr	0.412	0.0055769						
	TP/Cr	-0.335	0.0202564						
day10-12	SG	-0.533	0.0002949	WaxCrank	0.539	7.038E-06			
	NAG/Cr	0.408	0.0039694	NAG/Cr	0.471	6.483E-05			
				SG	-0.290	0.0061959			
				Alb/Cr	0.220	0.0429138			
day13-15	SG	-0.395	0.0028169	SG	-0.389	0.0013896	SG	-0.349	0.0108194
	RTErank	-0.290	0.0261747	CRP	0.419	0.000787	max severity	0.332	0.0149551
	sex	0.259	0.0443523	DM	0.369	0.0026122			
				L-FABP/Cr	-0.228	0.0489848			
day16-18	U-Na	0.824	0.0002691	GraCrank	0.185	0.187915	L-FABP/Cr	0.667	0.000175
	eGFR	-0.670	0.0022261	SG	0.584	6.389E-05	HyaCrank	-0.338	0.0193684
	DM	-0.392	0.0159816	eGFR	-0.390	0.0036446	D-Dimer	-0.357	0.0230437
				αMG/Cr	0.416	0.0051364			
day19-40	sex	0.404	0.0030337	TP/Cr	0.632	3.182E-06	αMG/Cr	0.698	1.625E-08
	eGFR	0.568	0.001072				eGFR	0.337	0.0017536
	NGAL/Cr	0.359	0.0277459				GraCrank	0.433	5.381E-05
	HyaCrank	0.271	0.0420077				WaxCrank	-0.237	0.0176165
All	SG	-0.312	2.055E-06	TP/Cr	0.302	0.0003079	αMG/Cr	0.648	3.735E-19
	U-Na	0.291	6.753E-06	NAG/Cr	0.251	0.002548	CRP	0.183	0.0012823
	max severity	0.237	0.0002113	HT	0.163	0.0067152	pH	0.136	0.0136289
	sex	0.176	0.0053907						

B

	Sph			dhSph		
	variables	β	P value	variables	β	P value
day1-6	D-Dimer	1.055	3.929E-10	D-Dimer	0.570	0.002381
	Alb/Cr	0.239	0.0035251			
	NAG/Cr	0.215	0.0077535			
	smoking	-0.265	0.0121133			
day7-9	GraCrank	0.507	0.0024689	smoking	0.519	0.0010861
	DM	0.366	0.0227319	αMG/Cr	0.419	0.0062296
day10-12	αMG/Cr	0.452	0.0003716	HT	-0.580	0.0004575
	SG	-0.343	0.0014109	pH	-0.354	0.0232331
	WaxCrank	0.403	0.0012292	smoking	0.301	0.0336849
	HT	-0.306	0.0039122			
day13-15	Alb/Cr	0.703	5.972E-07	max severity	0.342	0.0034077
	L-FABP/Cr	-0.541	9.606E-05	NGAL/Cr	0.408	0.0016271
	SG	-0.288	0.0085714	NAG/Cr	-0.269	0.0235922
	αMG/Cr	0.269	0.0223255			
day16-18	Alb/Cr	0.483	0.0055088	RTECrank	0.401	0.0280138
	RTErank	0.438	0.0109954			
day19-40	sex	0.732	9.109E-14	sex	0.490	0.0006399
	L-FABP/Cr	0.372	4.733E-06			
	U-Na	-0.266	0.0013302			
	SG	0.218	0.0053714			
All	NAG/Cr	0.628	5.535E-16	max severity	0.299	5.921E-06
	L-FABP/Cr	0.315	2.513E-07	HT	-0.227	0.0004333
	pH	-0.180	0.0006397	eGFR	0.195	0.0031451
	TP/Cr	-0.246	0.0008946	αMG/Cr	0.176	0.0081642

Clinical data
CRP, D-Dimer, max severity
eGFR
Urinary sediment findings
Chemical markers
SG, Urine sodium, pH

Supplemental Figure S15. The independent effects of clinical parameters on the urinary levels of lipids. The independent effects of the clinical properties and the results of urinary laboratory tests on urinary lipid levels were evaluated with a stepwise multiple regression analysis, using urinary lipid levels as objective variables and clinical information, maximum severity, eGFR, CRP, D-Dimer, urinary chemical markers, urinary sediment findings, SG, pH, and urinary sodium levels as potential explanatory factors. β represents the standardized coefficients.

Supplemental Figure S16

A

	C16:0 Cer			C18:0 Cer			C18:1 Cer		
	variables	β	P value	variables	β	P value	variables	β	P value
day1-6	αMG/Cr	0.672	2.464E-05	αMG/Cr	0.536	0.0048026	NAG/Cr	0.576	0.0001087
	SG	-0.381	0.0066642				HyaCrank	0.543	0.0010865
day7-9	L-FABP/Cr	0.414	0.0164069	smoking	0.685	0.000401	NAG/Cr	0.564	2.544E-06
	NAG/Cr	0.382	0.0255234	max severity	0.377	0.012192	RTErank	0.506	1.911E-05
				D-Dimer	-0.354	0.0406723	U-Na	0.733	6.12E-07
							D-Dimer	-0.376	0.0015066
day10-12	NGAL/Cr	0.765	8.233E-09	NGAL/Cr	0.569	0.0002895	Alb/Cr	0.564	9.72E-08
	HT	-0.318	0.0031352				NAG/Cr	0.522	3.786E-07
							SG	-0.206	0.014436
day13-15	NAG/Cr	0.413	0.0014937	U-Na	0.368	0.0109921	sex	0.366	0.0115141
	RTECrank	0.292	0.0177699						
	age	0.252	0.0472926						
day16-18				D-Dimer	0.579	0.000277	CRP	0.455	0.006784
				HyaCrank	-0.332	0.0239445	WaxCrank	0.334	0.0406956
day19-40	TP/Cr	0.920	6.299E-16	NAG/Cr	0.830	3.626E-05	NAG/Cr	0.681	2.595E-13
	NAG/Cr	0.819	1.013E-10	TP/Cr	0.454	0.0016844	sex	0.363	1.3E-06
	αMG/Cr	-0.817	8.541E-10	αMG/Cr	-0.395	0.048018	CRP	0.134	0.0196879
All	TP/Cr	1.752	1.513E-33	NAG/Cr	0.299	5.652E-05	NAG/Cr	0.375	8.138E-09
	NAG/Cr	0.454	1.321E-15	TP/Cr	0.406	1.769E-07	sex	0.162	0.0097967
	αMG/Cr	-0.553	3.126E-15	D-Dimer	0.194	0.0002898	SG	-0.154	0.0145906
	GraCrank	-0.071	0.0120944						

B

	C20:0 Cer			C22:0 Cer			C24:0 Cer		
	variables	β	P value	variables	β	P value	variables	β	P value
day1-6	NAG/Cr	0.576	0.0001087	NAG/Cr	0.582	0.0004346	NAG/Cr	0.563	0.0013853
	HyaCrank	0.543	0.0010865	HyaCrank	0.411	0.0081903	HyaCrank	0.329	0.0444671
day7-9	smoking	0.623	0.0004036	smoking	0.628	0.0001053	smoking	0.673	2.914E-05
				NAG/Cr	0.417	0.0053218	GraCrank	0.396	0.0071172
							NAG/Cr	0.339	0.0153245
day10-12	HT	-0.480	0.0014265	NGAL/Cr	0.505	0.0005441	HT	-0.422	0.0054303
	NGAL/Cr	0.393	0.0074694	HT	-0.432	0.002485	NGAL/Cr	0.412	0.0064485
day13-15	HyaCrank	0.500	0.0026775				NAG/Cr	0.510	0.0001665
	GraCrank	-0.665	0.0005287				smoking	-0.267	0.0368818
	RTECrank	0.456	0.0054562						
day16-18	smoking	0.388	0.0341462	TP/Cr	0.424	0.0195708	TP/Cr	0.472	0.0084175
day19-40	Alb/Cr	0.588	7.022E-11	NAG/Cr	0.498	3.998E-05	NAG/Cr	0.508	7.047E-10
	αMG/Cr	0.514	3.953E-09	Alb/Cr	0.229	0.0026848	Alb/Cr	0.528	2.638E-10
			4.521E-05	αMG/Cr	0.350	0.0046208			
All	αMG/Cr	0.430	1.99E-08	αMG/Cr	0.448	1.608E-07	αMG/Cr	0.316	9.34E-05
	TP/Cr	0.409	1.236E-07	NAG/Cr	0.312	6.86E-05	TP/Cr	0.403	8.204E-11
	age	0.144	0.0042501	Alb/Cr	0.171	0.00093	NAG/Cr	0.242	0.0005062

Clinical data
CRP, D-Dimer, max severity
eGFR
Urinary sediment findings
Chemical markers
SG, Urine sodium, pH

Supplemental Figure S16. The independent effects of clinical parameters on the urinary levels of lipids (continued). The independent effects of the clinical properties and the results of urinary laboratory tests on urinary lipid levels were evaluated with a stepwise multiple regression analysis, using urinary lipid levels as objective variables and clinical information, maximum severity, eGFR, CRP, D-Dimer, urinary chemical markers, urinary sediment findings, SG, pH, and urinary sodium levels as potential explanatory factors. β represents the standardized coefficients.

Supplemental Figure S17

A

	Total LPC			Total LPS			Total LPE		
	variables	β	P value	variables	β	P value	variables	β	P value
day1-6	eGFR	-0.550	0.0012259	NGAL/Cr	0.986	6.789E-22	NGAL/Cr	0.622	0.0007016
	HT	0.406	0.012175	HyaCrank	-0.065	0.0234944			
day7-9	Alb/Cr	0.708	2.461E-05	RTECrank	0.611	0.0005524	RTErank	0.698	5.268E-06
							sex	-0.357	0.0068948
day10-12	smoking	0.396	0.0101077	sex	1.442	2.506E-12	HT	-0.397	0.0164648
	NGAL/Cr	0.331	0.0289534	WaxCrank	-1.296	3.442E-11	RTErank	0.330	0.0435502
	pH	0.311	0.0390393	CRP	0.373	3.997E-05			
day13-15	age	0.377	0.0075874	GraCrank	0.782	0.0001132	sex	0.326	0.0252452
	DM	0.278	0.0449748	HyaCrank	-0.400	0.0169253			
day16-18	RTErank	0.535	0.0022975	L-FABP/Cr	0.550	0.0016397	SG	0.454	0.0072095
							eGFR	-0.416	0.0132433
							RTErank	0.334	0.0390109
day19-40	Alb/Cr	0.783	2.661E-19	TP/Cr	0.555	8.525E-08	L-FABP/Cr	2.134	1.489E-16
	NAG/Cr	0.348	6.805E-08	L-FABP/Cr	0.222	0.0002954	WaxCrank	-0.401	0.0003114
	WaxCrank	0.291	0.000341	αMG/Cr	0.304	0.0009809	GraCrank	-0.183	0.0010818
All	TP/Cr	0.645	2.468E-27	TP/Cr	0.341	3.723E-07	L-FABP/Cr	0.609	1.854E-22
	max severity	0.167	0.0015878				RTECrank	-0.124	0.0269868
	CRP	-0.126	0.0188591						
	RTErank	0.107	0.0406912						

B

	Total LPG			Total LPI			Total LPA		
	variables	β	P value	variables	β	P value	variables	β	P value
day1-6	D-Dimer	0.838	4.637E-09	RTErank	0.497	0.0097361	NGAL/Cr	0.584	4.682E-05
	NAG/Cr	0.395	0.0002111				U-Na	0.508	0.0002362
day7-9	sex	0.270	0.0071032	not selected			smoking	0.602	0.0007019
day10-12	smoking	0.512	0.0014297	NAG/Cr	0.491	0.0023432	max severity	0.386	0.0102154
							SG	-0.308	0.0366462
day13-15	DM	0.391	0.0068534	D-Dimer	0.543	3.298E-07	NAG/Cr	0.387	0.0054068
	U-Na	-0.278	0.0495131	CRP	0.518	6.617E-07	age	0.284	0.0370416
				HyaCrank	-0.220	0.0196696			
day16-18	GraCrank	0.568	0.0006823	L-FABP/Cr	0.563	0.0015043	Alb/Cr	0.633	3.163E-05
	CRP	0.448	0.0039441	DM	-0.332	0.0466294	U-Na	0.552	0.0009579
	WaxCrank	-0.452	0.0045939				age	0.384	0.0199283
day19-40	RTErank	0.639	5.266E-08				NGAL/Cr	0.544	5.992E-05
	NGAL/Cr	0.405	5.638E-05				DM	-0.307	0.011873
	HyaCrank	-0.182	0.0436501				SG	-0.259	0.0200092
All	L-FABP/Cr	0.195	0.0042734	D-Dimer	0.250	0.0002426	NGAL/Cr	0.306	3.168E-06
	DM	0.172	0.0111309				U-Na	0.252	0.0001897
	SG	-0.148	0.028254				age	0.175	0.0082069
							SG	-0.144	0.0290754

Clinical data
CRP, D-Dimer, max severity
eGFR
Urinary sediment findings
Chemical markers
SG, Urine sodium, pH

Supplemental Figure S17. The independent effects of clinical parameters on the urinary levels of lipids (continued). The independent effects of the clinical properties and the results of urinary laboratory tests on urinary lipid levels were evaluated with a stepwise multiple regression analysis, using urinary lipid levels as objective variables and clinical information, maximum severity, eGFR, CRP, D-Dimer, urinary chemical markers, urinary sediment findings, SG, pH, and urinary sodium levels as potential explanatory factors. β represents the standardized coefficients.

Supplemental Figure S18

A

	Total PC			Total PE			Total PG		
	variables	β	P value	variables	β	P value	variables	β	P value
day1-6	D-Dimer	1.064	3.495E-11	NGAL/Cr	0.768	4.4E-12	L-FABP/Cr	0.681	0.0006506
	pH	0.352	0.0002813	D-Dimer	0.488	1.483E-08	U-Na	0.466	0.0010766
	DM	-0.341	0.0004282	NAG/Cr	0.264	0.0001281	CRP	-0.326	0.0348463
	sex	0.183	0.0291086				max severity	0.310	0.0493755
day7-9	max severity	0.532	0.0009089				U-Na	0.609	0.0003523
	RTECrank	-0.408	0.0045271				αMG/Cr	0.376	0.0172649
	NAG/Cr	0.295	0.0466571						
day10-12	SG	-0.545	0.0001062	NGAL/Cr	0.341	0.0319263	NAG/Cr	0.483	0.001515
	NAG/Cr	0.472	0.0005803	SG	-0.338	0.0333007	SG	-0.379	0.0105036
day13-15	SG	-0.422	0.0013688	NGAL/Cr	0.488	0.0011336	SG	-0.575	3.147E-05
	sex	0.269	0.035179	L-FABP/Cr	-0.365	0.0130945	U-Na	0.316	0.0140446
	RTErank	-0.269	0.0369577	SG	-0.298	0.0260639	sex	0.247	0.0453956
day16-18	U-Na	0.953	2.184E-05	αMG/Cr	0.443	0.0020859	HyaCrank	-0.465	0.0083224
	eGFR	-0.701	0.0006909	WaxCrank	0.368	0.0079634	smoking	-0.339	0.0473575
	DM	-0.464	0.0038667	NGAL/Cr	0.349	0.0107596			
	Alb/Cr	0.311	0.0386744						
day19-40	sex	0.396	0.0071253	TP/Cr	0.540	1.211E-12	NAG/Cr	0.840	3.444E-06
				NAG/Cr	0.448	1.556E-10	Alb/Cr	-0.474	0.0059969
				HyaCrank	-0.155	4.393E-05	pH	0.326	0.0087003
				U-Na	-0.106	0.0031471	HyaCrank	0.271	0.0248749
All	SG	-0.356	1.635E-08	TP/Cr	1.297	1.358E-21	NAG/Cr	0.337	2.035E-08
	NAG/Cr	0.450	1.264E-07	SG	-0.195	2.393E-05	SG	-0.347	1.682E-08
	U-Na	0.228	0.0002258	RTECrank	-0.139	0.0020936	U-Na	0.311	2.879E-07
	TP/Cr	-0.269	0.0012524						

B

	Total PI			Total PS		
	variables	β	P value	variables	β	P value
day1-6	L-FABP/Cr	0.735	1.159E-05	smoking	0.684	4.887E-06
	RTErank	-0.349	0.0144597	NAG/Cr	0.292	0.019212
				HT	0.264	0.0322806
day7-9	max severity	0.445	0.0093702	RTErank	0.636	0.0035958
	U-Na	0.377	0.024795	RTECrank	-0.461	0.028267
day10-12	NAG/Cr	0.519	9.973E-05	smoking	0.426	0.0073106
	SG	-0.397	0.0014012	NGAL/Cr	0.344	0.0272621
	TP/Cr	0.284	0.0215976			
day13-15	SG	-0.419	0.000592	D-Dimer	0.363	0.0121082
	sex	0.376	0.0016869			
	CRP	0.355	0.0029628			
day16-18	L-FABP/Cr	1.246	3.329E-05	CRP	0.711	1.488E-06
	NAG/Cr	-0.759	0.0053991	NGAL/Cr	0.413	0.0011692
				RTErank	0.301	0.012716
				SG	0.280	0.0204061
day19-40	NAG/Cr	0.777	3.618E-10	TP/Cr	0.921	1.266E-23
				sex	0.216	1.705E-05
All	NAG/Cr	0.569	1.583E-21	TP/Cr	1.268	1.834E-16
	SG	-0.290	1.431E-07	HyaCrank	-0.158	6.377E-05
	U-Na	0.161	0.0030652	NAG/Cr	0.144	0.0192543
	RTErank	-0.138	0.0106085	smoking	0.087	0.0255306

Clinical data
CRP, D-Dimer, max severity
eGFR
Urinary sediment findings
Chemical markers
SG, Urine sodium, pH

Supplemental Figure S18. The independent effects of clinical parameters on the urinary levels of lipids (continued). The independent effects of the clinical properties and the results of urinary laboratory tests on urinary lipid levels were evaluated with a stepwise multiple regression analysis, using urinary lipid levels as objective variables and clinical information, maximum severity, eGFR, CRP, D-Dimer, urinary chemical markers, urinary sediment findings, SG, pH, and urinary sodium levels as potential explanatory factors. β represents the standardized coefficients.

Supplemental Figure S19

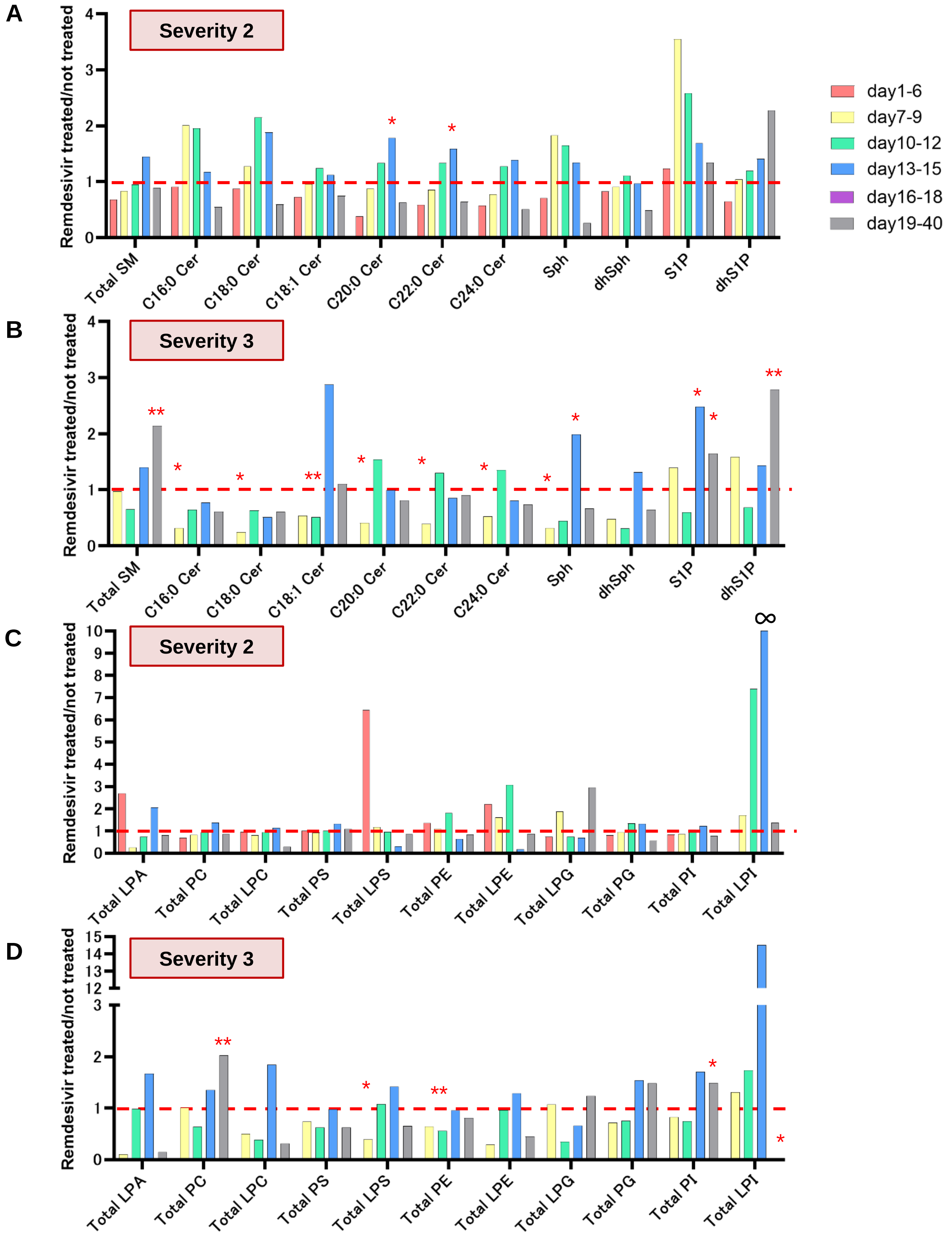

Supplemental Figure S19. The difference of urinary lipid levels between the subjects treated with remdesivir and those without. The urinary lipid levels were compared between the subjects treated with remdesivir and those without in a specific maximum severity group. The Mann-Whitney U test was used to evaluate the differences. The ratio of the urinary lipid levels in the subjects treated with remdesivir to those without were shown. *$p < .05$, **$p < .01$.

Supplemental Figure S20

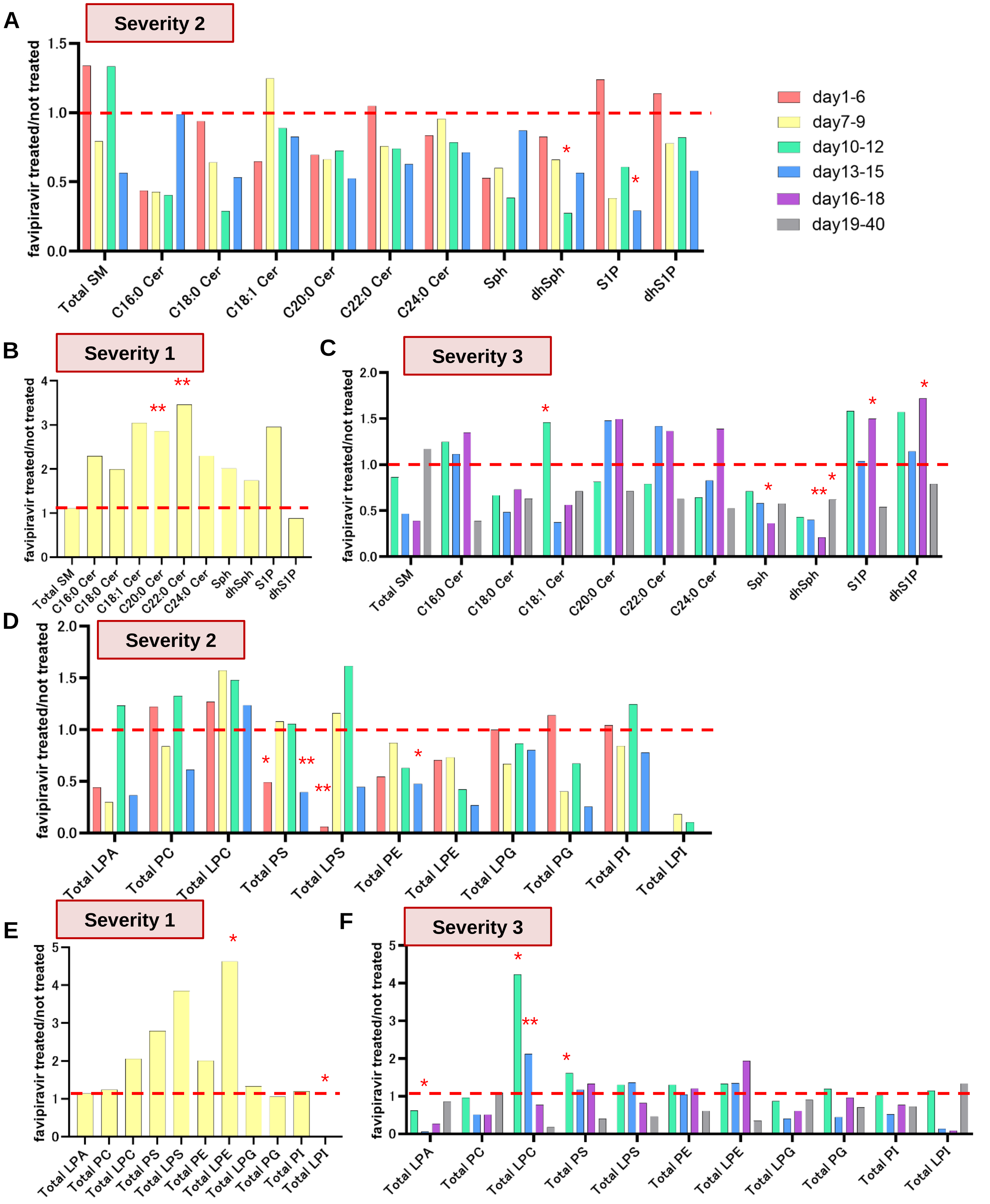

Supplemental Figure S20. The difference of urinary lipid levels between the subjects treated with favipiravir and those without. The urinary lipid levels were compared between the subjects treated with favipiravir and those without in a specific maximum severity group. The Mann-Whitney U test was used to evaluate the differences. The ratio of the urinary lipid levels in the subjects treated with favipiravir to those without were shown. *$p < .05$, **$p < .01$.

Supplemental Figure S21

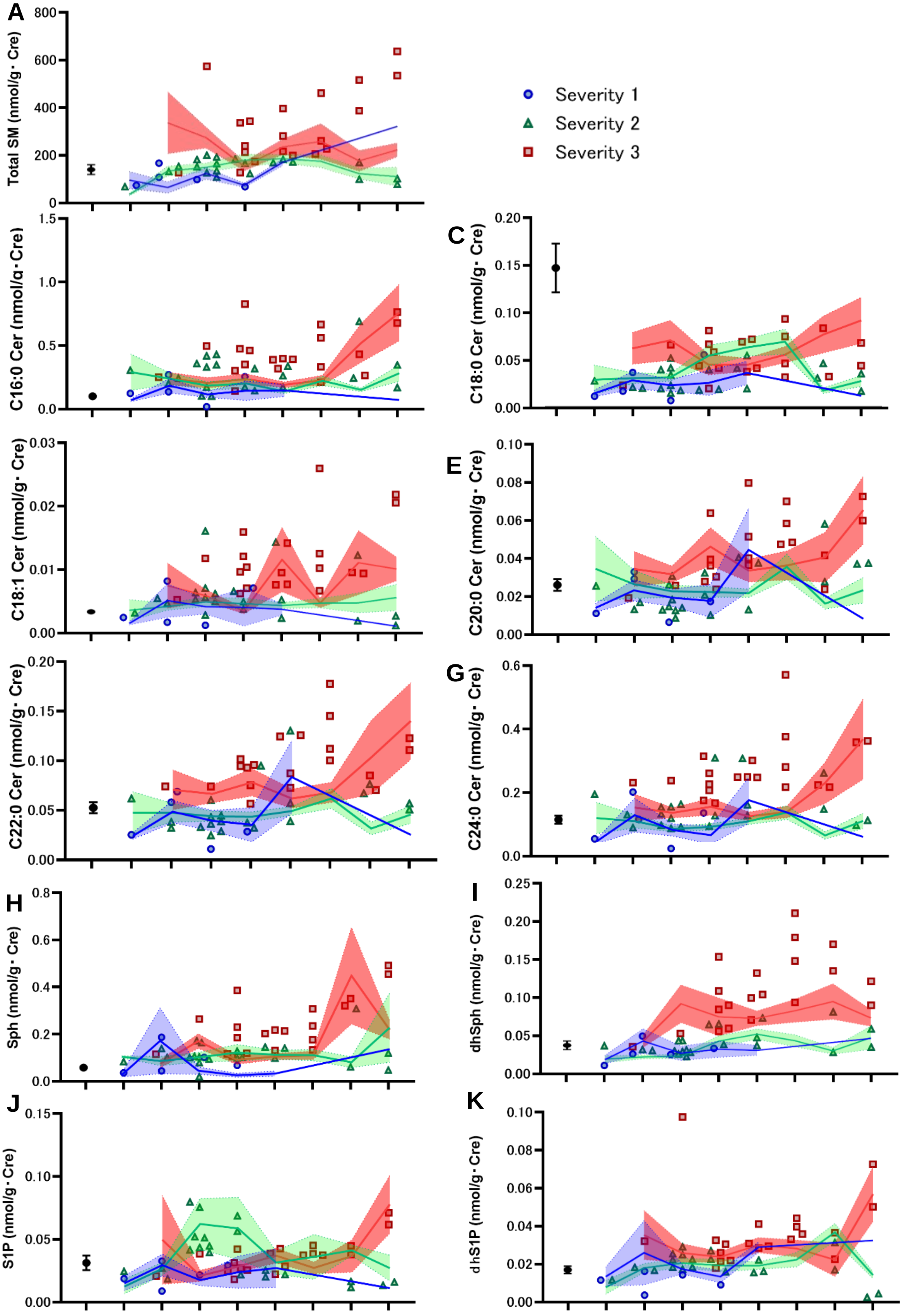

Supplemental Figure S21. The urinary levels of sphingolipids in independent samples were overlayed on Figure 1 To validate the results of the present study, we measured urinary sphingolipid levels in independent urine samples and the results were overlayed on Figure 1.

Supplemental Figure S22

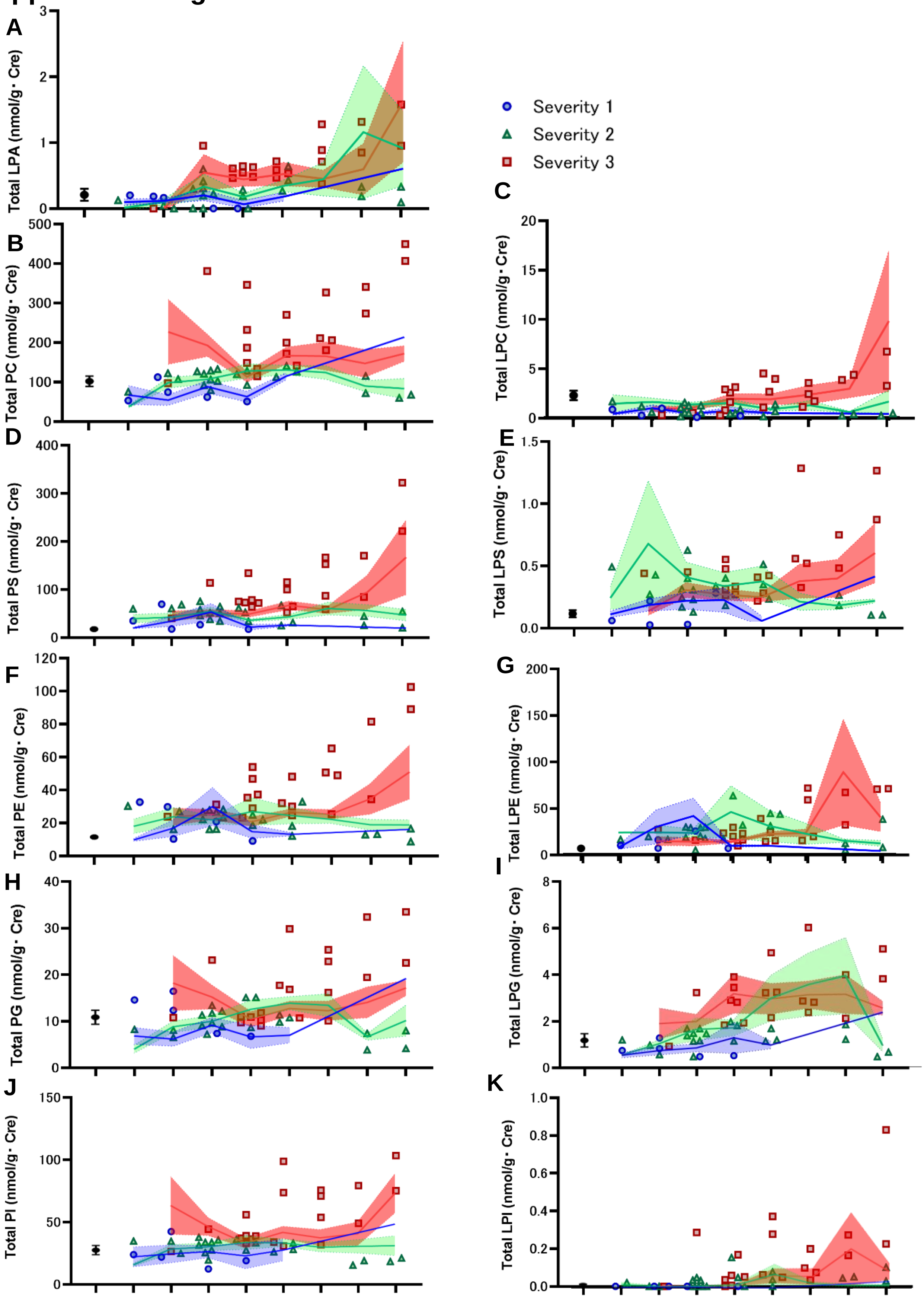

Supplemental Figure S22. The urinary levels of glycerophospholipids in independent samples were overlayed on Figure 2 To validate the results of the present study, we measured urinary glycerophospholipid levels in independent urine samples and the results were overlayed on Figure 2.

Supplemental Figure S23

PC, LPA, LPC

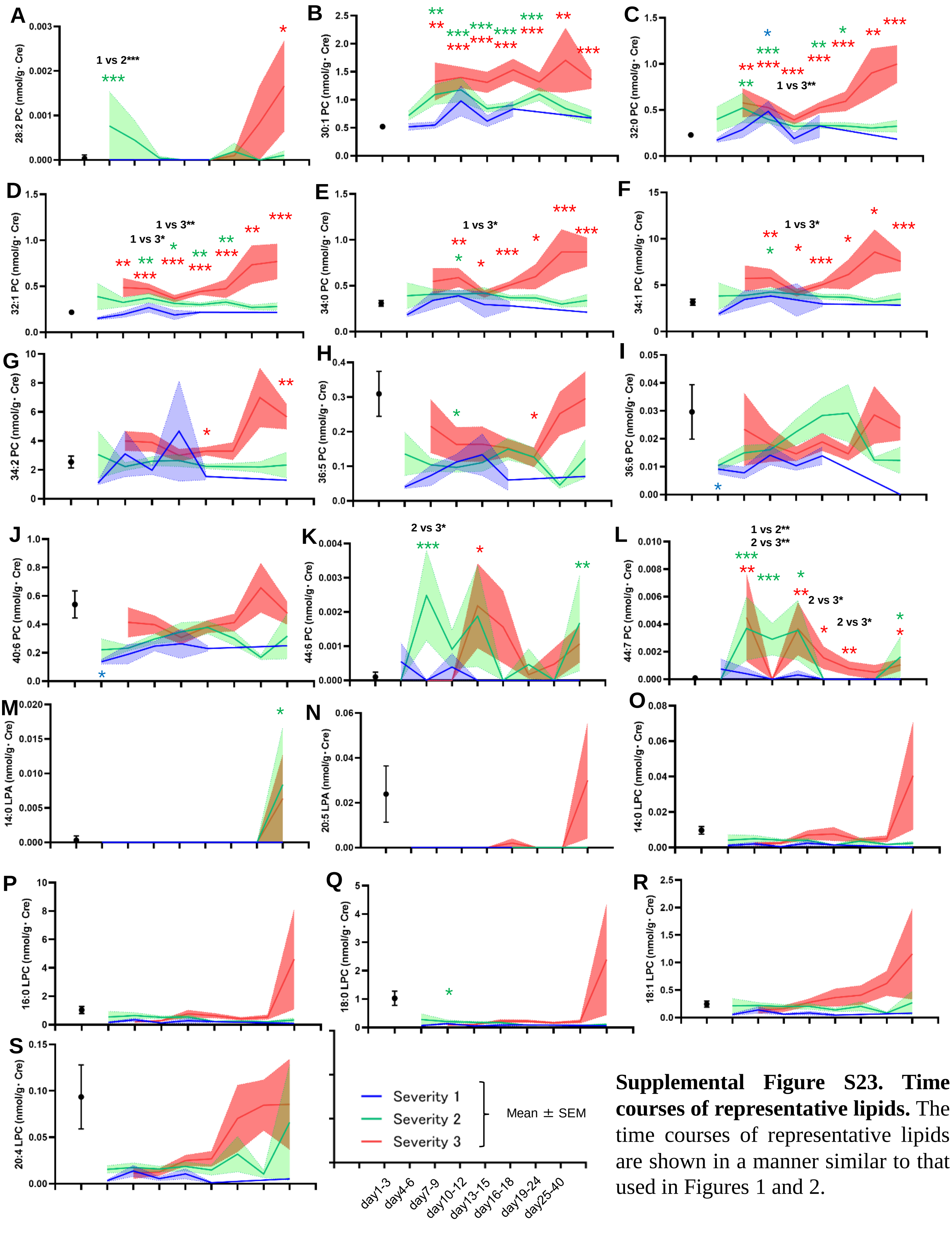

Supplemental Figure S23. Time courses of representative lipids. The time courses of representative lipids are shown in a manner similar to that used in Figures 1 and 2.

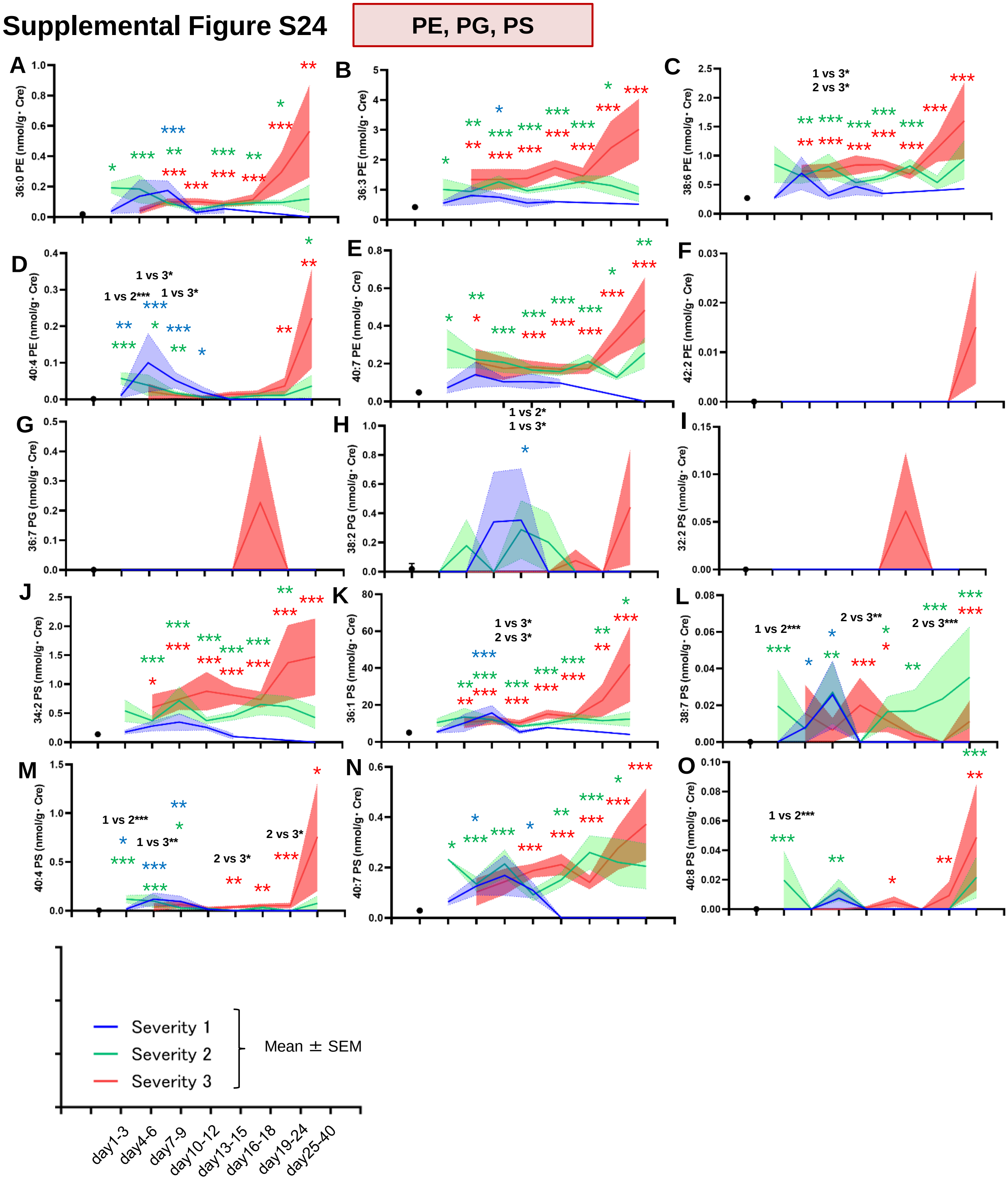

Supplemental Figure S24. Time courses of representative lipids (continued). The time courses of representative lipids are shown in a manner similar to that used in Figures 1 and 2.

Supplemental Figure S25

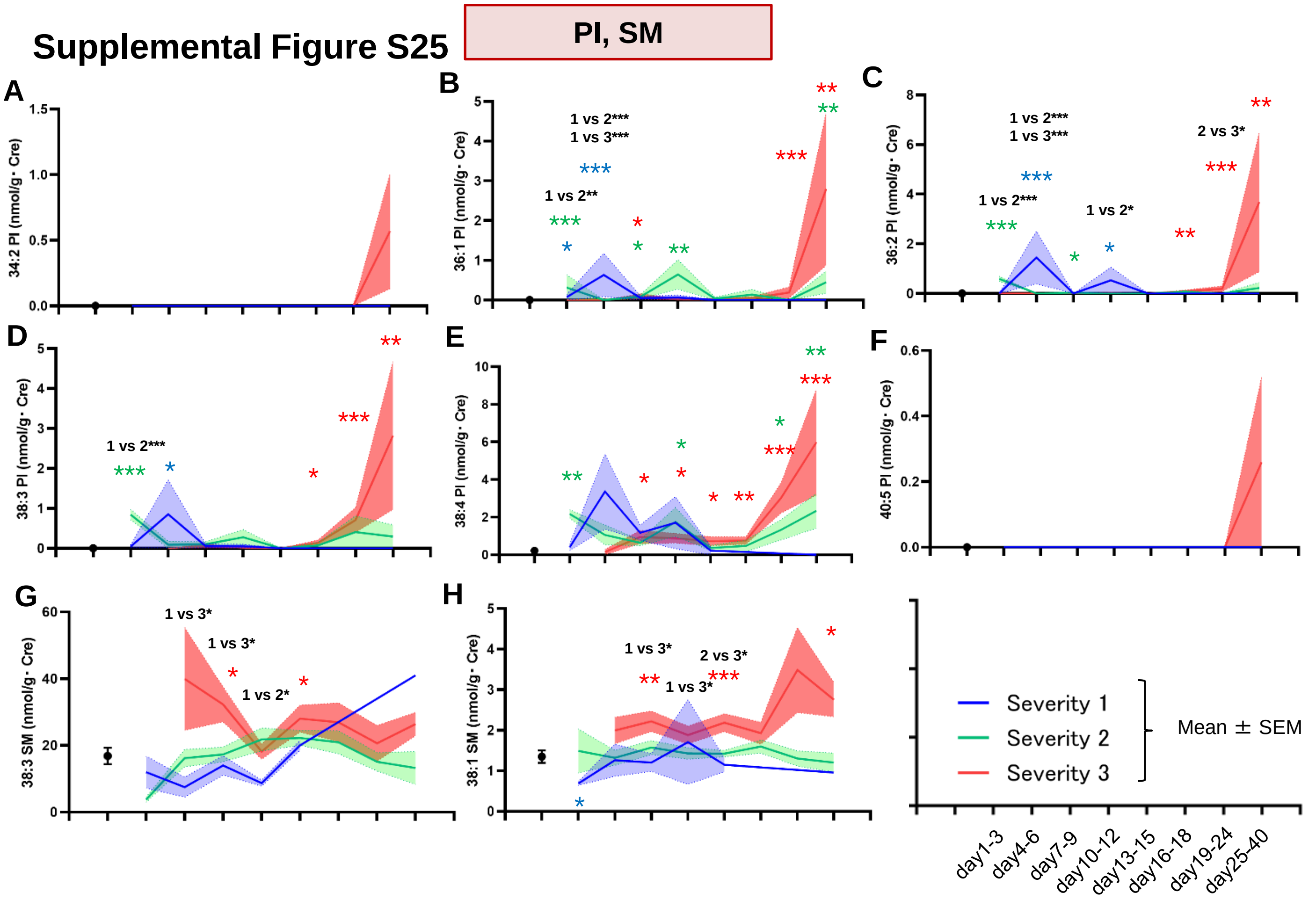

Supplemental Figure S25. Time courses of representative lipids (continued). The time courses of representative lipids are shown in a manner similar to that used in Figures 1 and 2.

Supplemental Figure S26

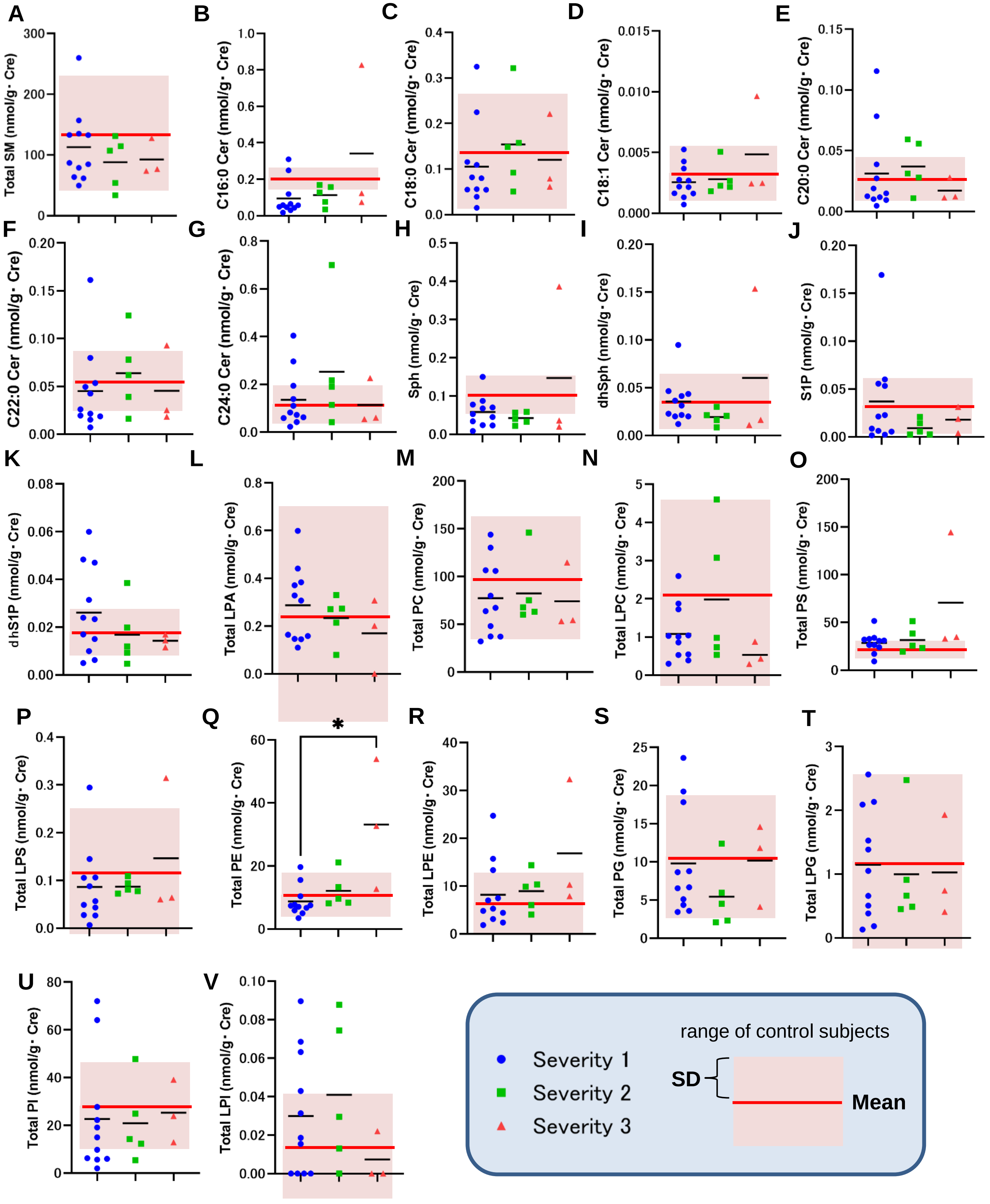

Supplemental Figure S26. The urinary levels of lipids in the post-COVID-19 subjects after more than two months from the onset. The urinary levels of lipids were measured in the urine samples collected from the post-COVID-19 subjects after more than two months from the onset. Maximum severity group 1 (n = 11), maximum severity group 2 (n = 5), and maximum severity group 3 (n = 3). The difference was evaluated using the Steel-Dwass test followed by the Kruskal-Wallis test. *$p < .05$.
